# Identification and expression profiling of the *CoDof* genes involved in fatty acid/lipid biosynthesis of tetraploid *Camellia oleifera*

**DOI:** 10.3389/fpls.2025.1599849

**Published:** 2025-06-09

**Authors:** Guang Zhao, Lina Chen, Lin Zhang, Chancan Liao, Jin Wang

**Affiliations:** ^1^ Key Laboratory of Cultivation and Protection for Non-Wood Forest Trees, Ministry of Education, Central South University of Forestry and Technology, Changsha, China; ^2^ Hunan Forestry Seedling Breeding Demonstration Center, The Forestry Department of Hunan Province, Changsha, China

**Keywords:** tetraploid *Camellia oleifera*, *CoDof* gene family, fatty acids/lipids, expression, *CoDof30.1*

## Abstract

**Introduction:**

*Camellia oleifera*, a crucial woody oil crop in China, produces seeds with over 90% unsaturated fatty acids offering substantial nutritional value and exists predominantly as cultivated tetraploid varieties (2n=4x=60) due to its polyploid nature. The DNA-binding with one finger (Dof) transcription factor play multiple roles in plant growth, development, and abiotic stress response pathways. However, the regulatory mechanisms of *Dof* genes underlying fatty acids/lipids biosynthesis during seed morphogenesis in *Camellia oleifera* remain poorly characterized.

**Methods:**

In this study, genome-wide identified a total of 40 members of the *CoDof* family with 116 alleles in tetraploid *Camellia oleifera* (COL-tetra).

**Results:**

All members possess varying numbers of highly conserved C_2_-C_2_-type zinc finger domains. Phylogenetic analysis clustered *CoDof* genes into nine categories, and significant divergence was observed in the expression levels of all family members across different growth and development stages of COL-tetra seeds. After physiological data determination at various levels, differential expression analysis and correlation analysis of fatty acid/lipid synthesis genes revealed that *CoDof30.1* is a typical candidate nuclear-localized transcription factor which significantly highly expressed in the middle period of seed development.

**Discussion:**

Our findings not only comprehensively characterize the genomic organization of *CoDof* family but also propose a functional candidate for lipid biosynthesis regulation, thereby advancing molecular breeding strategies and elite cultivar selection in COL-tetra.

## 1 Introduction

Driven by the exponentially growing global demand for premium-grade lipids and their value-added derivatives, investigations into plant-based fatty acid biosynthesis and lipid metabolic pathways have emerged as a pivotal research frontier in agricultural biotechnology. Tea oil tree (*Camellia oleifera*), a woody oilseed crop native to East Asia, commonly known as the ‘king plant’, is renowned for its high-quality seed oil rich in monounsaturated fatty acids (MUFAs), particularly oleic acid (C18:1). The oil, widely used in food, cosmetics, and traditional medicine, has gained global attention due to its health benefits and industrial applications (Yang et al., 2024). Tetraploid *Camellia oleifera* (COL-tetra) cultivars, developed through polyploidization, exhibit superior traits such as increased biomass, larger seeds, and higher oil content compared to diploid counterparts, with great ecological and economic significance (Zhang et al., 2024).

Fatty acid and lipid biosynthesis constitute central metabolic pathways that enable plants to dynamically allocate carbon and nitrogen resources while adapting to environmental challenges (Ohlrogge and Browse, 1995). These interconnected biochemical processes not only fulfill essential roles in energy storage (primarily through triacylglycerol biosynthesis) and membrane biogenesis, but also produce specialized metabolites critical for developmental regulation and stress adaptation (Nagaoka et al., 2021). Fatty acids are categorized into straight-chain fatty acids (SCFAs), branched-chain fatty acids (BCFAs), and ring fatty acids (RFAs). SCFAs are the primary source of energy, synthesized through a pathway involving key genes such as CIT, SREBP, and PPAR, which regulate energy metabolism and fat storage. The biosynthesis of SCFAs involves iterative condensation of acetyl-CoA and malonyl-CoA by fatty acid synthase (FAS), with elongation and reduction steps encoded by genes like FASN in eukaryotes (Shimano and Sato, 2017).

In contrast, BCFA production diverges at the precursor level through the incorporation of branched-chain α-ketoacid dehydrogenase (BCKDH) products, notably isovaleryl-CoA and isobutyryl-CoA (Yoneshiro et al., 2019). The BCKDH multienzyme complex, encoded by BCKDHA and BCKDHB genes, generates these methyl-branched starter units that are subsequently elongated by FAS machinery to produce iso/anteiso-15:0 and related structures. Genetic studies in *Arabidopsis* demonstrate that BCFA-deficient mutants exhibit compromised cuticular barrier function and enhanced susceptibility to necrotrophic pathogens (Xia et al., 2010). The synthesis of RFAs involves HMG-CoA synthase, which produces the precursor for these specialized lipids. RFAs form through cyclization of unsaturated intermediates, such as cyclopropane rings added by cyclopropane fatty acid synthase (Cfa) encoded by *cfa* in bacteria, or via polyketide synthase pathways for complex ring systems in plants and fungi, with structural diversity governed by modular synthase genes (Austin and Noel, 2003). Additionally, structural genes such as *FAS*, *Fasn*, *Nes*, *HMG-CoA synthase*, *VitaminD*, *SLC22A3*, *GluPr*, and *Tarch* play critical roles in lipid metabolism, including phospholipid synthesis, cholesterol production, and class II lipid synthesis. These genes are essential for regulating energy metabolism, signaling pathways, and cellular functions (Smith et al., 2003).

*The DNA-binding with one finger* (*Dof*) transcription factor family represents a plant-specific group of transcriptional regulators characterized by a conserved C_2_-C_2_-type zinc finger domain containing four cysteine residues. As a distinct subclass within the zinc finger superfamily, Dof proteins typically consist of 200–400 amino acids with a bipartite domain architecture: a highly conserved N-terminal DNA-binding domain and a divergent C-terminal transcriptional regulatory domain (Noguero et al., 2013). The conserved N-terminal domain (~52 amino acids) adopts a unique single zinc finger structure stabilized by a CX2CX21CX2C motif, where one Zn^²+^ ion is tetrahedrally coordinated by four conserved cysteine residues (Cys-X2-Cys-X21-Cys-X2-Cys) (Noguero et al., 2013). This metal-binding configuration is functionally crucial, as evidenced by the complete loss of DNA-binding activity upon treatment with divalent ion chelators or site-directed mutagenesis of cysteine residues (Sharif et al., 2012). The Dof proteins exhibit sequence-specific DNA recognition capability, primarily binding to the AAAG core motif in target gene promoters to regulate transcriptional processes. Through combinatorial interactions with other transcriptional regulators, these proteins participate in diverse physiological processes including photoperiodic flowering, seed maturation, vascular development, and abiotic stress responses (Kim et al., 2010). Notably, the C-terminal regulatory domain shows considerable sequence divergence across family members, which has been proposed as the molecular basis for functional diversification within this transcription factor family. This structural plasticity enables Dof proteins to act as key integrators in plant signaling networks, mediating crosstalk between hormonal pathways (particularly auxin and gibberellin signaling) and environmental stimuli (Corrales et al., 2017).

The Dof protein family represents a group of ubiquitous transcription factors in higher plants, exhibiting distinct species-specific expansion patterns. Comparative genomic analyses have revealed 36, 31, 30, and 54 *Dof* family members in *Arabidopsis thaliana* (Yanagisawa, 1997), wheat (Noguero et al., 2013), rice (Lijavetzky et al., 2003), and maize (Zhang et al., 2023), respectively. These zinc finger-containing transcriptional regulators play pivotal roles in coordinating plant growth and developmental processes. In *Arabidopsis*, constitutive overexpression of *OBP1* (*OBF-BINDING PROTEIN 1*), a *Dof* transcription factor, induces significant morphological alterations through coordinated changes in cell proliferation and expansion, ultimately resulting in dwarf phenotypes (Skirycz et al., 2008). Similarly, rice *OsDof12* gene participates in plant architecture formation, where its overexpression reduces transgenic rice plant height and decreases the number of primary and lateral branches (Wu et al., 2015). Furthermore, Dof proteins play critical roles in plant carbon-nitrogen metabolism and secondary metabolite biosynthesis. Notably, the ginseng (*Panax ginseng*) *PgDof14–1* gene exhibits significant co-expression with key enzyme genes involved in saponin biosynthesis, suggesting its potential regulatory function in ginsenoside production (Wei et al., 2018). Similarly, *AtDof4.7* and *AtDof5.6* in *Arabidopsis* have been demonstrated to modulate the expression of fatty acid synthesis-related genes (Wei et al., 2010). However, the functional characterization of *Dof* genes in fatty acid and lipid biosynthesis pathways remains unreported in *Camellia oleifera*.

In this study, systematic characterization of *CoDof* transcription factors in the COL-tetra genome revealed their multifaceted regulatory roles in fatty acid biosynthesis and lipid metabolic pathways. These findings not only establish a functional framework for *CoDof* gene investigations but also advance molecular breeding strategies for cultivar optimization and genetic marker development in COL-tetra.

## 2 Results

### 2.1 Genome-wide identification *CoDof* family in COL-tetra

Employing a hidden Markov model (HMM) profile corresponding to the Dof zinc-finger domain (PF02701), a HMMER3 search was performed on the COL-tetra database to identify potential *CoDof* candidate genes. The basic information of 40 *CoDof* family members, including 116 different gene isoforms. The alleles of all family members are paired and distributed between homologous chromosomes, and assign a *CoDof* ID to each gene based on chromosomal information and polyploidy homology. The number of amino acids of *CoDof* ranges from 84 (*CoDof7.2*) to 373 (*CoDof17.2/CoDof18.2/CoDof21.2*), with corresponding molecular weights varying from 9251.21 (*CoDof7.2*) to 41652.62 (*CoDof18.2*). The theoretical pI values show a wide distribution, from 4.72 (CoDof22.3) to 9.64 (CoDof13.2), indicating different acid-base properties. The instability index, aliphatic index, and grand average of hydropathicity are also provided. The instability index ranges from 26.58 (CoDof28.3) to 73.96 (CoDof17.3), reflecting the stability of the proteins. The aliphatic index varies between 33.08 (CoDof37/CoDof38.4/CoDof38.5) and 129.88 (CoDof7.2), and the grand average of hydropathicity ranges from -1.216 (CoDof17.1) to 0.351(CoDof7.2), which are related to hydrophobicity and structural characteristics of the protein. It is showed that except for CoDof7.2, the remaining 39 DoDof proteins all have a total average hydrophobicity index less than 1, indicating that most members of the Dof family are hydrophilic proteins. In addition, the physical and chemical properties of each allele are very similar, indicating that the functional genes in COL-tetra are relatively conserved (**Supplementary Table S1**).

### 2.2 Conserved domains analysis of *CoDof* gene in COL-tetra

**Figure 1:**
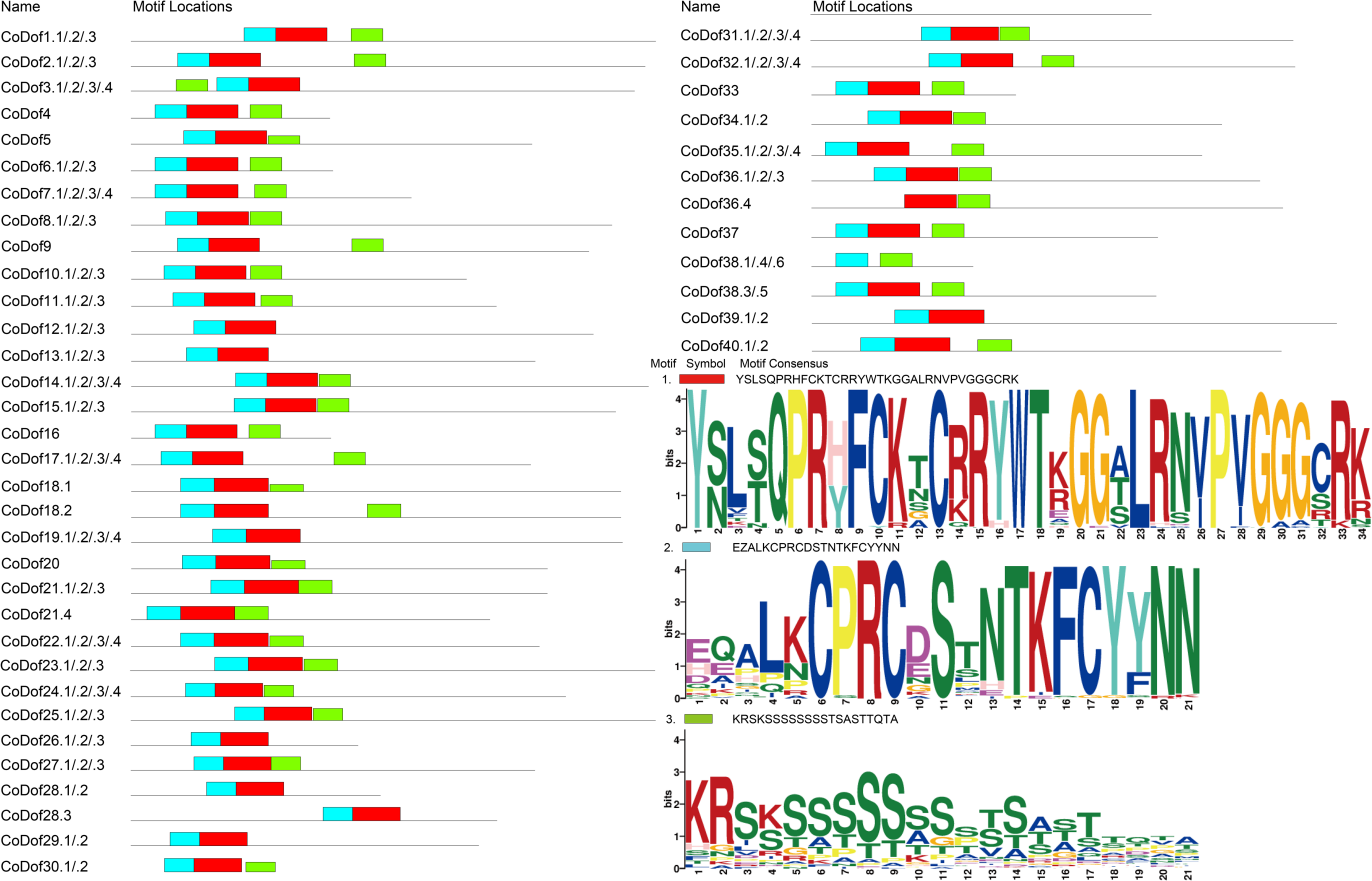
Conserved domains of the *CoDof* gene family. The distributions of conserved motifs in *CoDof*s, orange bars represent as motif 1, blue bars represent as motif 2, and green bars represent as motif 3.

The MEME web-based platform was used to analysis 40 CoDof proteins with a total of 116 allele sequences uncovered three distinct conserved motifs. Three motifs displayed lengths ranging from 21 to 35 amino acid residues, and the variation in motif composition (number and type) across different CoDof proteins suggests functional diversification *in vivo*, possibly due to their distinct biological roles. Protein sequence alignment results show that the amino acid sequences in the characteristic domain regions of CoDof proteins exhibit relatively strong conservation, with three distinct forms: motif1 (GGCRK, indicated in red), motif2 (CPRCD, indicated in blue) and motif3 (indicated in green). In the characteristic domain of Dof proteins, a total of 28 amino acids are the most conserved, which may play a key role in contributing to the conservation of the Dof domain. Within the characteristic region of Dof proteins, there is one conserved motif (motif1) and one highly conserved C_2_-C_2_ type zinc finger structure (CX2CX21CX2C, Zinc-finger). The C_2_-C_2_ type zinc finger structure is located within the conserved motif (motif2). The four conserved Cys residues are an important feature of the zinc finger structure and a key characteristic for identifying the Dof domain. Furthermore, the conserved motif analysis of CoDof proteins shows that the amino acid sequences of all members contain motif1, which is located within the characteristic domain of the CoDof family, implying the conservation of the characteristic Dof domain among family members. Conserved motifs in CoDof members of the same equipotential exhibit a certain degree of similarity in composition and arrangement. However, it is particularly noted that CoDof38.1, CoDof38.4, and CoDof38.6 lack the conserved motif 2, whereas CoDof38.3 and CoDof38.5 possess the conserved motif 2 structure (**Figure 1**).

### 2.3 Gene structure analysis of *CoDof* member in COL-tetra

**Figure 2:**
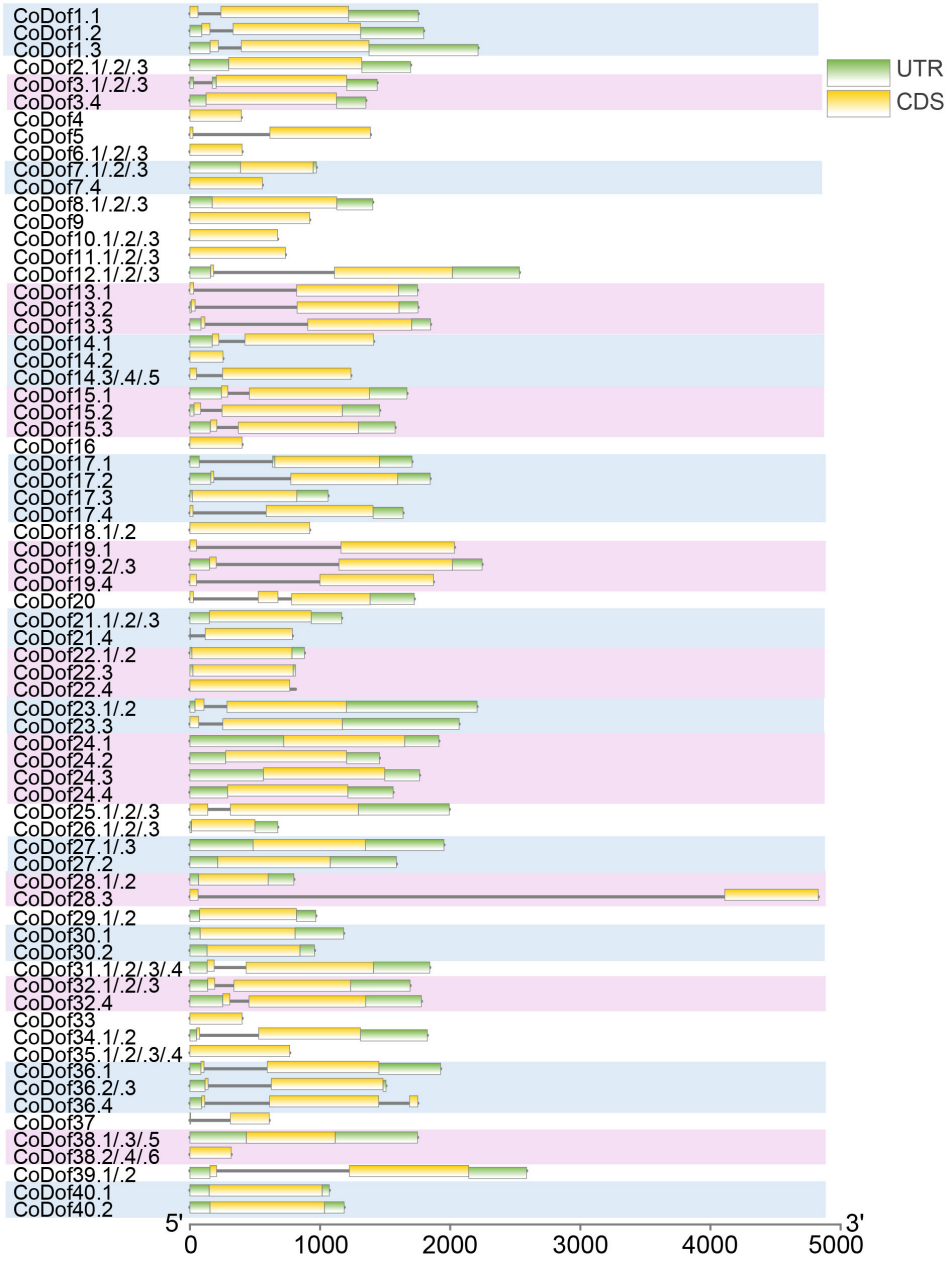
Gene structure of the *CoDof* gene family. The horizontal axis represents the length in base pairs, ranging from 0 to 5000, while the vertical axis lists different *CoDof* members, some of which have multiple variants (e.g., *CoDof1.1*, *CoDof1.2*). The yellow bars represent exon, the black lines represent intron, and green bars represent UTR regions.

Gene structure analysis revealed that the number of introns of the *CoDof* family members is between 0 and 2, There are 62 *CoCofs* have no introns, 52 *CoDofs* contain one intron, and only 2 genes contain two introns, namely *CoDof20* and *CoDof36.4*. Besides, the lengths of UTRs and CDSs vary significantly among different members. For example, *CoDof28.3* has a relatively long CDS compared to others. Some members, like *CoDof1.1*, *CoDof1.2* and *CoDof1.3*, show different patterns of UTR and CDS lengths, indicating possible alternative splicing events. Different types of allele representation provide a comprehensive view of the structural diversity within the *CoDof* gene family (**Figure 2**).

### 2.4 Phylogenetic tree analyses for *CoDofs*

**Figure 3:**
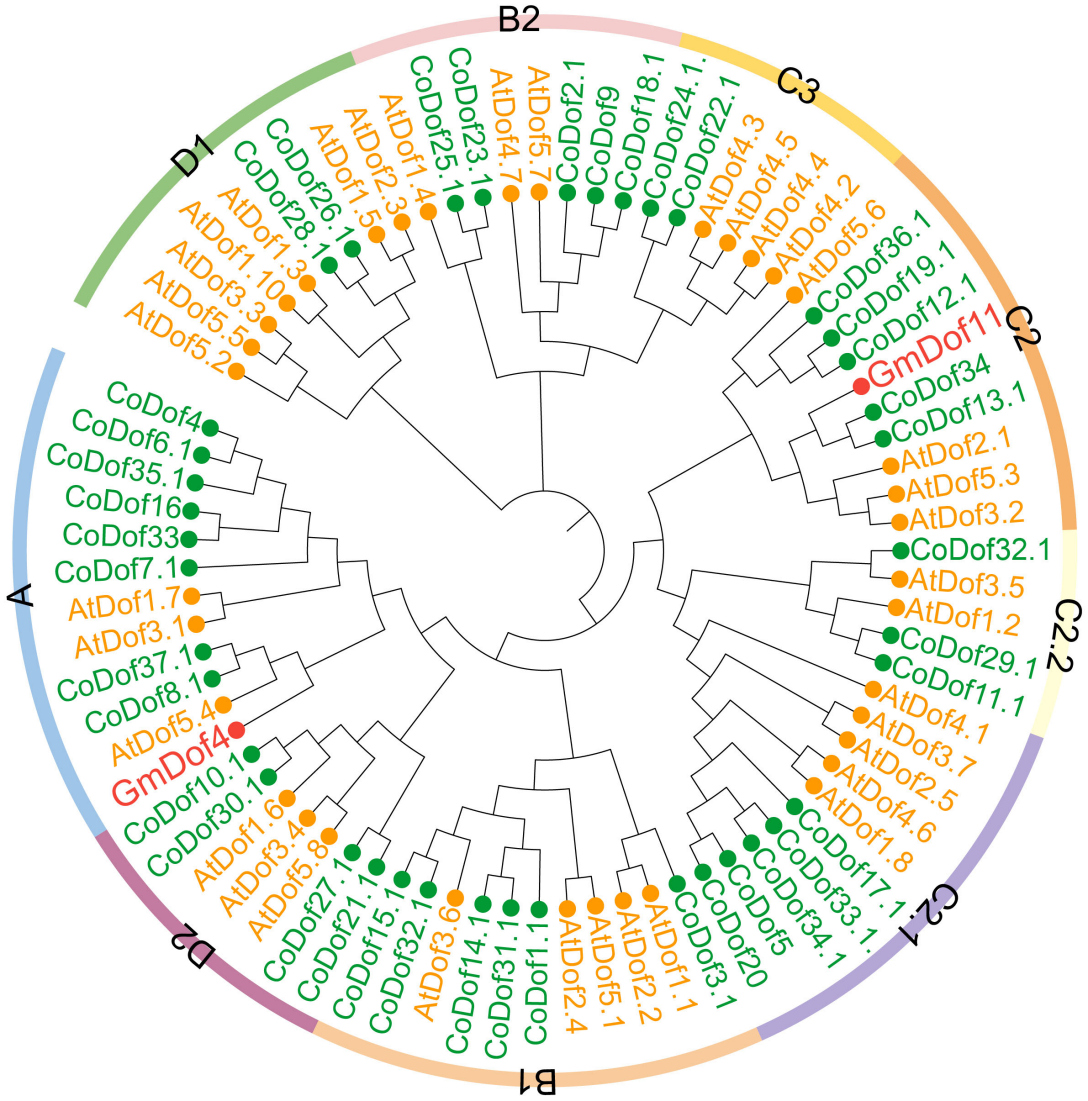
*Phylogenetic tree* of CoDofs and AtDofs proteins. Species are distinguished by name prefixes, AtDof (*Arabidopsis thaliana*), CoDof (COL-tetra), and GmDof (*Glycine max*). Different colored dots assist in distinguishing species origin. Maximum Likelihood (ML) method, with bootstrap testing conducted over 1000 replicates.

Phylogenetic tree cluster analysis of CoDof family members was performed using *Arabidopsis thaliana* and selected soybean GmDof family members as references. Based on *Arabidopsis* classification criteria, the Dof proteins was classified into 9 subfamilies, including A, B1, B2, C2, C2.1, C2.2, C3, D1, and D2 (**Figure 3**). Among them, the A subclass contained the highest number of CoDof family proteins (8 members), followed by the B1 subfamily with 6 DoDof members. Genes within the family exhibit sequence homology and functional similarity across plant species. GmDof4 and GmDof11, associated with fatty acid and lipid biosynthesis, were located in the A and C2 subfamilies respectively, indicating that proteins in these two subclades are linked to lipid metabolic processes. Furthermore, significant differences in distribution proportions of family members exist among different species within the same subfamily, reflecting functional divergence of Dof proteins across taxa (**Figure 3**).

### 2.5 Cis-elements analysis in the promoter of *CoDofs*

**Figure 4:**
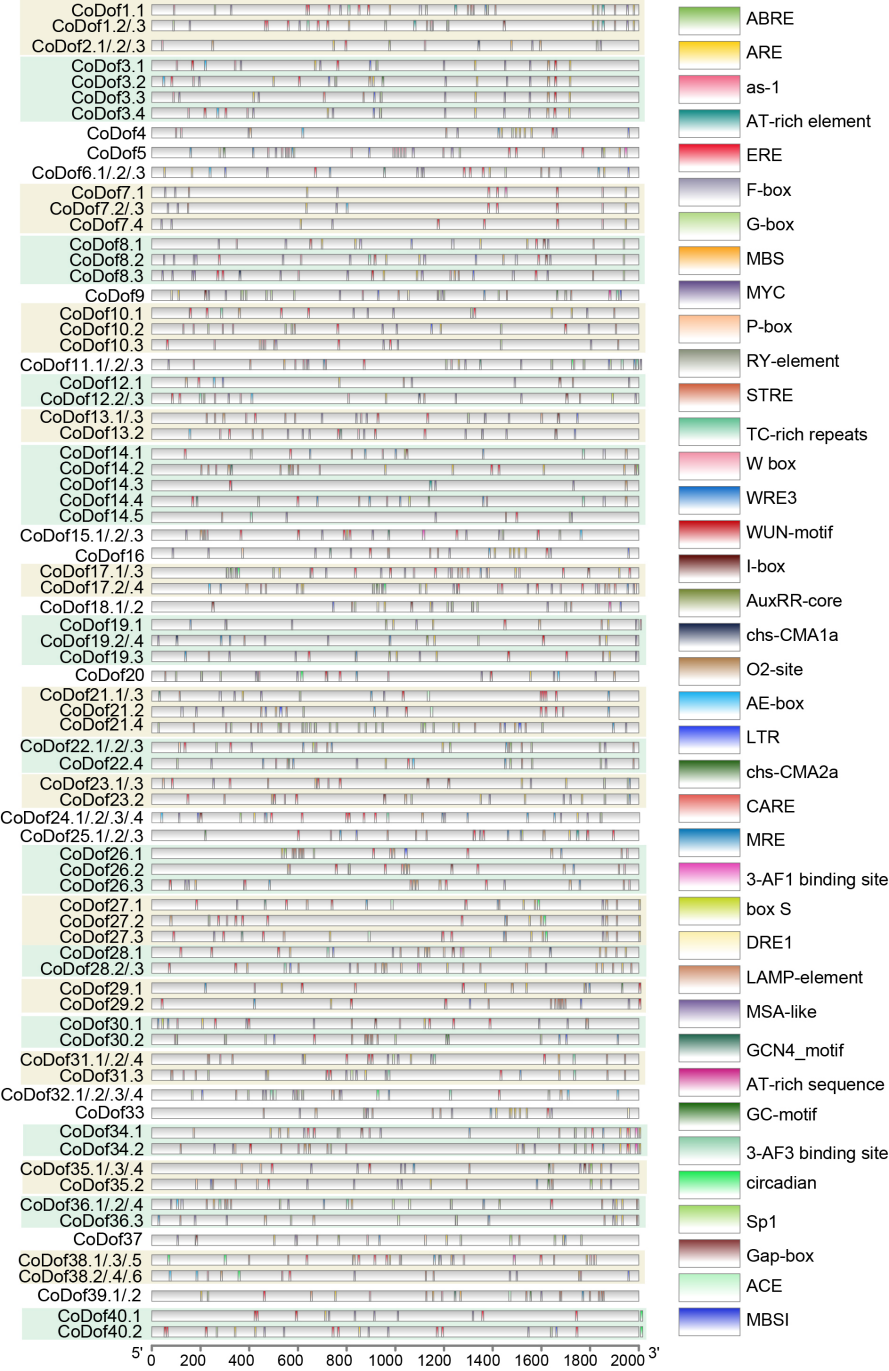
Predicted cis-elements analysis in the promoter regions of *CoDof* genes. Analysis of 2-kb upstream promoter regions for *CoDof*s genes was conducted using the PlantCARE platform to characterize cis-regulatory elements. Regulatory elements were visualized with distinct geometric symbols and color-coded representations according to functional categories. Detailed annotation of identified cis-elements is provided in **Supplementary Table S2**.

The 2-kilobase promoter regions upstream of *CoDof*s genes and the actin housekeeping gene were computationally analyzed using the PlantCARE database to identify potential cis-acting regulatory elements (**Figure 4**). This analysis revealed five categories of cell development-related cis-elements in *CoDof*s promoters, including endosperm expression, palisade mesophyll cells, flavonoid biosynthetic genes regulation, and cell cycle regulation motifs. Additionally, four hormone-responsive regulatory modules were identified, consisting of abscisic acid-, auxin-, and gibberellin -responsive elements, along with zein metabolism regulatory sequences. Notably, 20 stress-responsive cis-elements were characterized, encompassing light responsive elements, anaerobic induction, circadian control, anoxic specific inducibility, Ethylene-responsiveelement, elicitor-mediated activation, low-temperature responsiveness, drought-inducibility, defense and stress responsiveness, and wound-responsiveness (**Supplementary Table 2**). Conservation analysis of the actin promoter region identified core regulatory elements such as ABRE, ARE, ERE, G-box, MYC, and STRE, highlighting the conserved regulatory features across COL-tetra genes.

### 2.6 RNA-seq analysis of COL-tetra seeds in divergence stages

**Figure 5:**
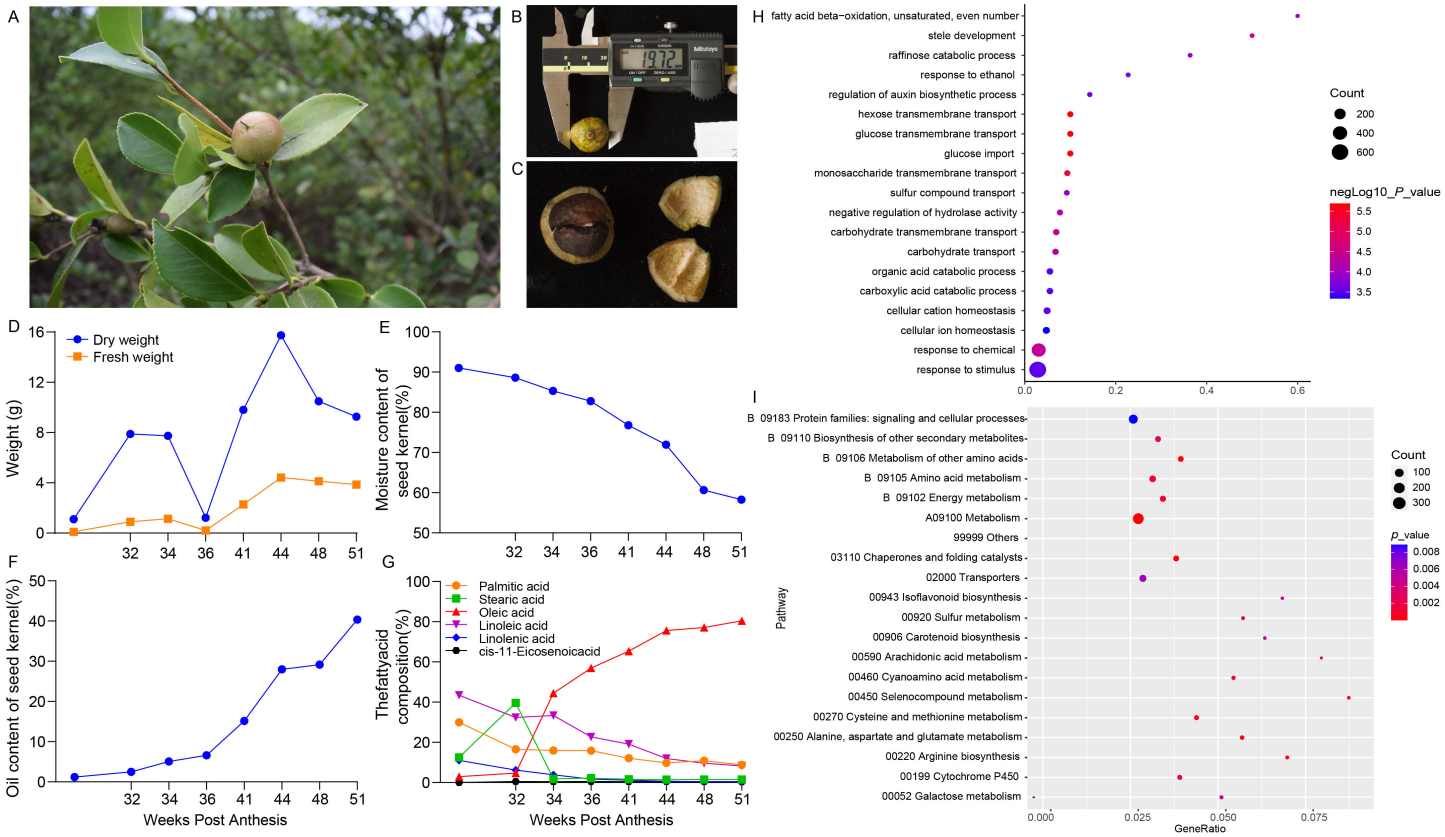
Phenotypic characteristics and physiological changes of COL-tetra seeds during development. **(A)** Phenotypic characteristics of COL-tetra seeds; **(B)** Measurement of seed diameter using a caliper, showing an average diameter of 19.12 mm; **(C)** Internal structure of the seed, including the seed coat and kernel; **(D)** Changes in dry and fresh weight of the fruits over time, with dry weight peaking at 12 weeks and fresh weight remaining relatively stable; **(E)** Decrease in moisture content of the seed kernel over time; **(F)** Increase in oil content of the seed kernel, reaching its highest level at 16 weeks; **(G)** Changes in fatty acid composition over time, with oleic and linoleic acid contents increasing while palmitic and stearic acid contents decrease; **(H)** Gene Ontology (GO) enrichment analysis; **(I)** Kyoto Encyclopedia of Genes and Genomes (KEGG) pathway enrichment analysis. The size and color of the dots represent the count and significance of the enrichment, respectively.

To study the expression pattern and pathway of *CoDof* genes in COL-tetra, we first sampled the seed tissue of COL-tetra and determined the fatty acid content. The phenotypic characteristics seeds showing the fruits as ellipsoidal with a smooth surface, transitioning in color from green to red (**Figure 5A**). The average diameter of the seeds is approximately 19.12 mm (**Figure 5B**), with the kernel being dark brown and hard in texture (**Figure 5C**). The physiological changes during fruit development illustrates the changes in dry and fresh weight over time, with dry weight increasing rapidly in the early stages, peaking at 34 weeks, and then slightly decreasing (**Figure 5D**). Fresh weight remains relatively stable throughout the development period. The moisture content of the seed kernel, which decreases steadily from the initial stages (**Figure 5E**). The oil content of the seed kernel, which increases gradually, reaching its highest level at 51 weeks (**Figure 5F**). The changes in fatty acid composition, with oleic and linoleic acid contents increasing over time, while palmitic and stearic acid contents decrease (**Figure 5G**).

Besides, transcriptome sequencing was performed using RNA-seq based on different developmental stages of seeds. According to the Gene Ontology (GO) enrichment results, the obtained differential expression genes (DEGs) were significant enriched in biological processes such as fatty acid beta-oxidation, unsaturated even-numbered fatty acid metabolic process, raffinose catabolic process, and regulation of auxin biosynthetic process. These results indicate that fatty acid metabolism and carbohydrate transport are crucial biological processes during fruit development (**Figure 5H**). The Kyoto Encyclopedia of Genes and Genomes (KEGG) revealed that the DEGs were significant enrichment in pathways related to signaling and cellular processes, biosynthesis of other secondary metabolites, and amino acid metabolism. These findings suggest that signal transduction and metabolic pathways play important roles in the development of COL-tetra seeds (**Figure 5I**).

### 2.7 Expression patterns of triacylglycerols and fatty acid synthesis genes in COL-tetra

**Figure 6:**
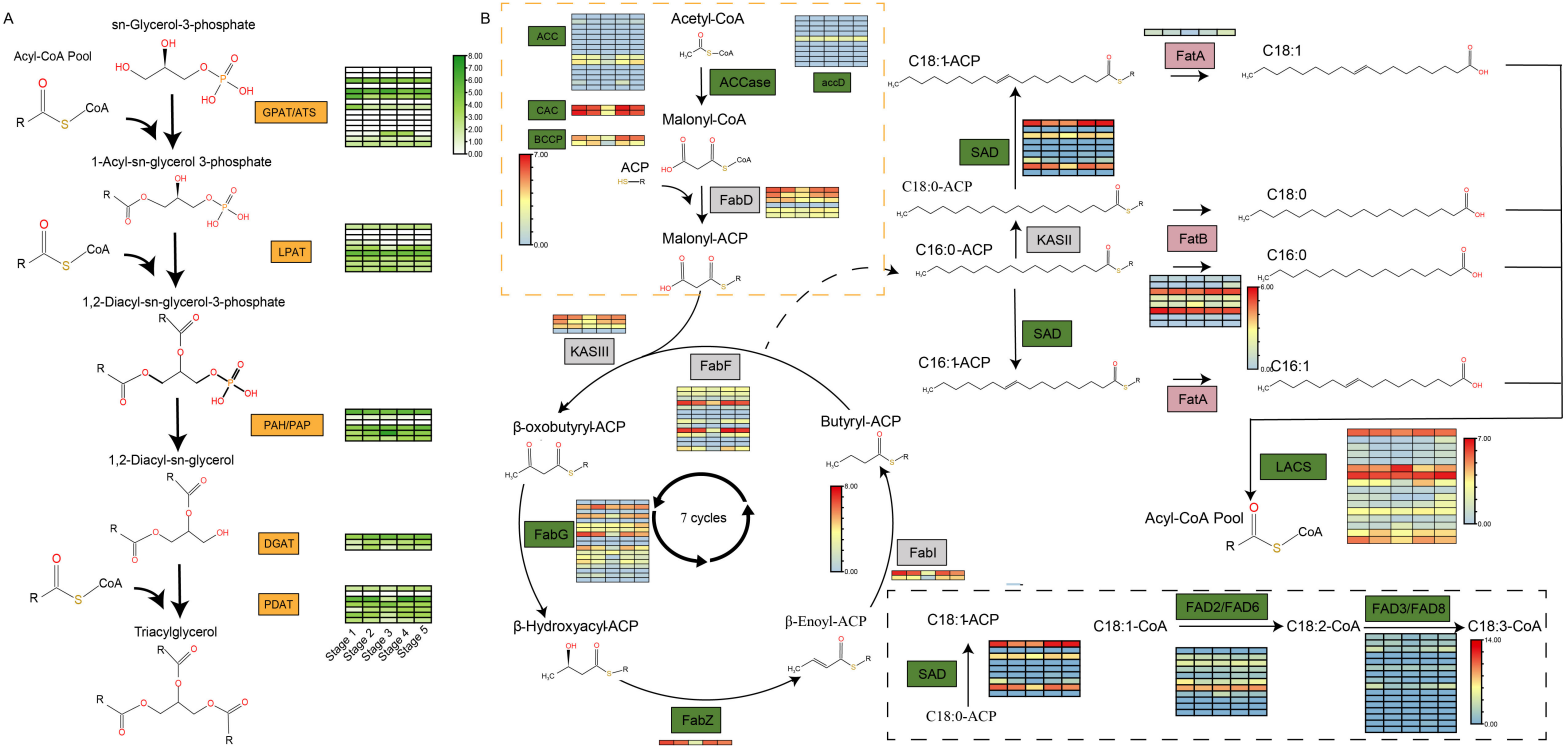
Analysis of triacylglycerol and fatty acid synthesis pathways and gene expression levels at different stages of seed development. **(A)** The triacylglycerol synthesis pathway of COL-tetra and gene expression analysis at different stages of seed development; **(B)** The fatty acid synthesis pathway of COL-tetra and gene expression analysis at different seed development stages.

The synthesis of triacylglycerols (TAG) in COL-tetra involves intricate enzymatic systems and diverse chemical reactions, primarily mediated through the acyl-CoA-dependent Kennedy pathway and the acyl-CoA-independent phospholipid: diacylglycerol acyltransferase (PDAT) pathway. A total of 13 *GPAT*, 1 *ATS*, 3 *PAP*, 3 *PAH*, 9 *LPAT*, 1 *DGAT1*, 2 *DGAT2*, and 7 *PDAT* genes were identified during TAG biosynthesis (**Figure 6A**). Analysis of their expression patterns across seed developmental stages revealed three key insights: first, not all members of the same gene family are transcriptionally active, as exemplified by only 6 of the 13 *GPAT* genes being expressed, suggesting selective silencing of certain family members during lipid synthesis; second, dynamic temporal regulation governs gene expression, with *PDAT1* exhibiting a fluctuating rise-fall-rise-fall pattern and *PDAT2a* displaying a simpler rise-fall trend, implying coordinated functional specialization within gene families; third, *DGAT1*, the terminal rate-limiting enzyme in TAG assembly, showed significantly higher expression than *DGAT2*, while PDAT pathway genes outnumbered and surpassed *DGAT* genes in both quantity and expression levels. This dual-pathway collaboration between PDAT and DGAT enzymes likely underpins the compositional diversity of COL-tetra lipids (**Figure 6A**).

Besides, the expression analysis of genes related to fatty acid biosynthesis in COL-tetra. For genes with multiple alleles, the expression levels of different alleles were summed. In the *de novo* fatty acid biosynthesis pathway, ACCase acts as a key rate-limiting enzyme. Multiple gene families encoding different subunits of ACCase were identified in COL-tetra. Expression profiling of these gene families during different seed developmental stages revealed that not all members of the same gene family were expressed - some family members remained silent during seed development. Additionally, expressed genes showed higher expression levels in early and late developmental stages compared to the third stage. However, seed fatty acid content continued to increase throughout development, with lower accumulation in early stages, suggesting that fatty acids synthesized in early stages were not fully converted into storage lipids. Multiple gene families involved in fatty acid chain elongation were also identified. Expressed genes within these families displayed similar expression patterns - higher in early and late stages, lower in mid-stage. Given the high oleic acid content (up to 80%) in COL-tetra oil, enzymes related to 18-carbon fatty acid desaturation were specifically analyzed. Nine *SAD* genes, 11 *FAD2/FAD6* genes, and 16 *FAD3/FAD8* genes were identified. Interestingly, despite oleic acid accounting for over 80% of total fatty acids (compared to ~8% linoleic acid and 0.5% linolenic acid), the gene family encoding stearoyl-ACP desaturase (SAD) showed the fewest members, while ω-3 fatty acid desaturase genes (FAD3/FAD8) were most abundant. This further confirms that gene copy number does not directly correlate with phenotypic traits. Expression analysis revealed significantly higher *SAD* gene expression across all developmental stages compared to *FAD2/FAD6*, which in turn showed higher expression than *FAD3/FAD8*. This expression hierarchy explains the characteristic high-oleic, low-linoleic, low-linolenic acid composition of COL-tetra oil (**Figure 6B**).

### 2.8 Expressions and correlation analyzed of *CoDofs* and fatty acid/lipid synthesis genes in divergence stages

**Figure 7:**
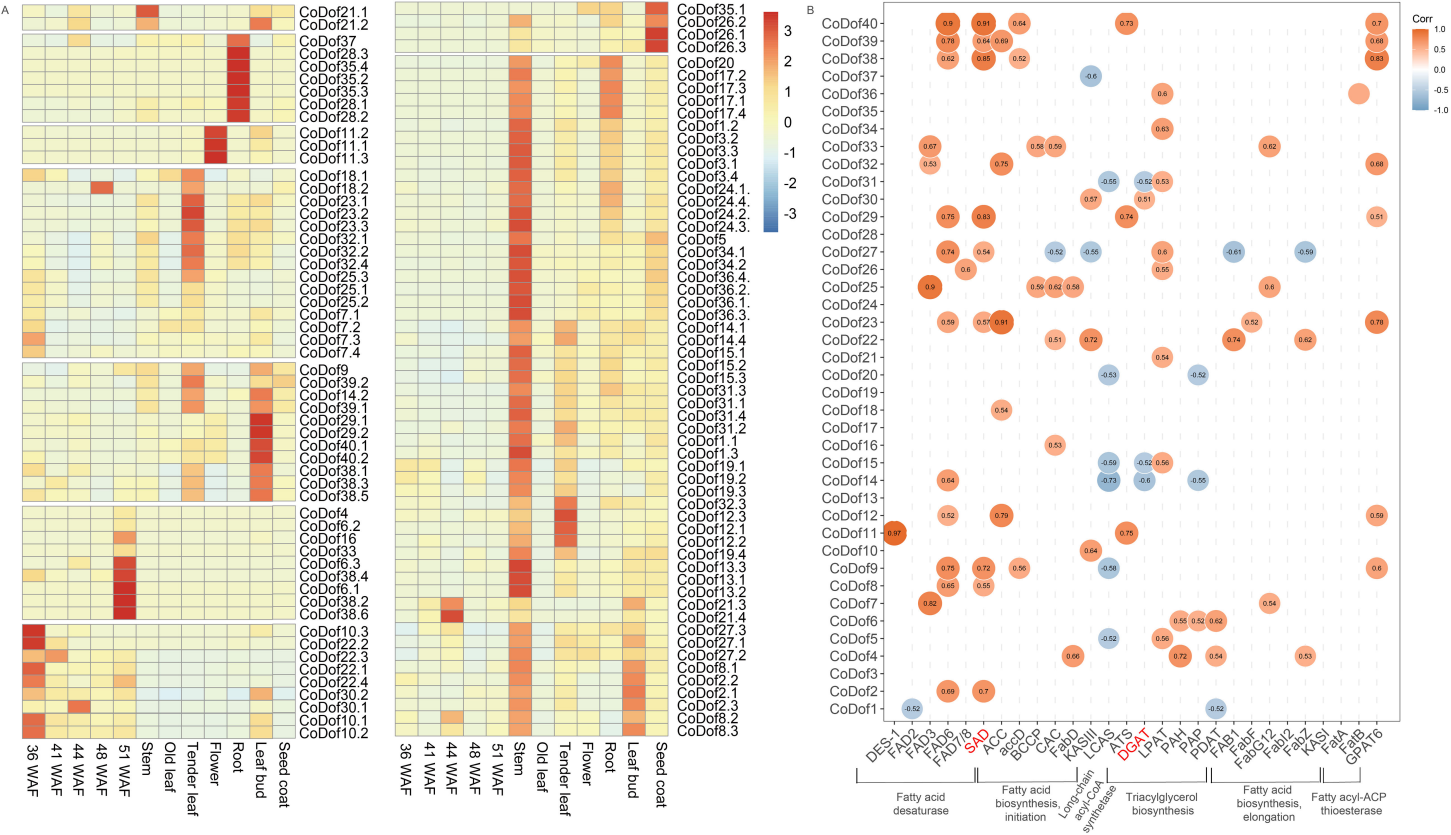
Expression and correlation analysis of *CoDof*s. **(A)** Expression profiles based on RNA-seq fragments per kilobase million (FPKM) data; **(B)** The Pearson’s correlation coefficient analysis was conducted between fatty acid/lipid biosynthesis-related genes and *CoDof* transcription factors. The intensity of association was proportional to the absolute value of the correlation coefficient, with significant relationships (|r| > 0.6) visualized in the diagram. Positive correlations were represented by orange shading, while negative relationships were denoted in blue.

To investigate the functional diversity of *CoDof* gene family members throughout the developmental cycle of COL-tetra, we conducted expression pattern analysis using RNA-seq BAM files from multiple organ systems and developmental phases. The heatmap visualization revealed distinct spatial-temporal expression profiles among *CoDof* members, enabling their classification into eight expression clusters (**Figure 7A**). Notably, three genes (*CoDof37*, *CoDof28*, and *CoDof35*) exhibited preferential expression in root tissues, while *CoDof11* demonstrated floral-specific activation. Two distinct expression patterns emerged in photosynthetic tissues, *CoDof18*, *CoDof23*, *CoDof32*, *CoDof25*, and *CoDof7* showed meristem-associated expression in tender leaves, whereas *CoDof9*, *CoDof39*, *CoDof14*, *CoDof29*, *CoDof40*, and *CoDof38* displayed dual activation in both tender leaves and leaf buds. Besides, seed developmental analysis uncovered stage-specific regulation: Five genes (*CoDof4*, *CoDof6*, *CoDof16*, *CoDof33*, and *CoDof38*) displayed late maturation-phase activation (16-week seeds), contrasting with four genes (*CoDof10*, *CoDof22*, *CoDof30*, and *CoDof10*) showing early developmental preference. Specialized expression patterns were observed for *CoDof35* and *CoDof26* in seed coat formation, while the remaining family members exhibited stem-enriched expression profiles. These differential expression patterns across developmental stages and tissue types suggest functional diversification of *CoDof* transcription factors in regulating growth and organogenesis processes in COL-tetra. The stage-specific activation of particular gene subsets implies specialized roles in temporal developmental regulation, while tissue-preferential expression indicates spatial functional differentiation within plant architecture.

Because on fatty acid/lipid pathways, Pearson correlation analysis was performed between fatty acid/lipid biosynthesis genes and *CoDofs* family members. The result revealed that *CoDof11.1, CoDof40.1* and *CoDof25.1* were strongly positively linked with fatty acid desaturase pathway, with correlation coefficient is greater than 0.9. The *CoDof25.1, CoDof33* and *CoDof7.1* were strongly positively linked with fatty acid biosynthesis initiation pathway, *CoDof30.1* and *CoDof22.1* was highly positively associated with long-chain acyl-CoA synthetase. Besides, the *CoDof30.1*, *CoDof6.2*, and *CoDof4* was highly positively associated with triacylglycerol biosynthesis, while *CoDof22.1* was strongly positively linked with fatty acid biosynthesis elongation pathway. Importantly, the *CoDof29*, *CoDof38*, *CoDof40*, and *CoDof30* as candidate transcription factors highly positively associated with *DGAT* and *SAD, which* play crucial roles in *de novo* biosynthesis of fatty acids and lipid (**Figure 7B**).

### 2.9 A candidate *CoDof30.1* gene may be involved in Fatty acid and lipid process

**Figure 8:**
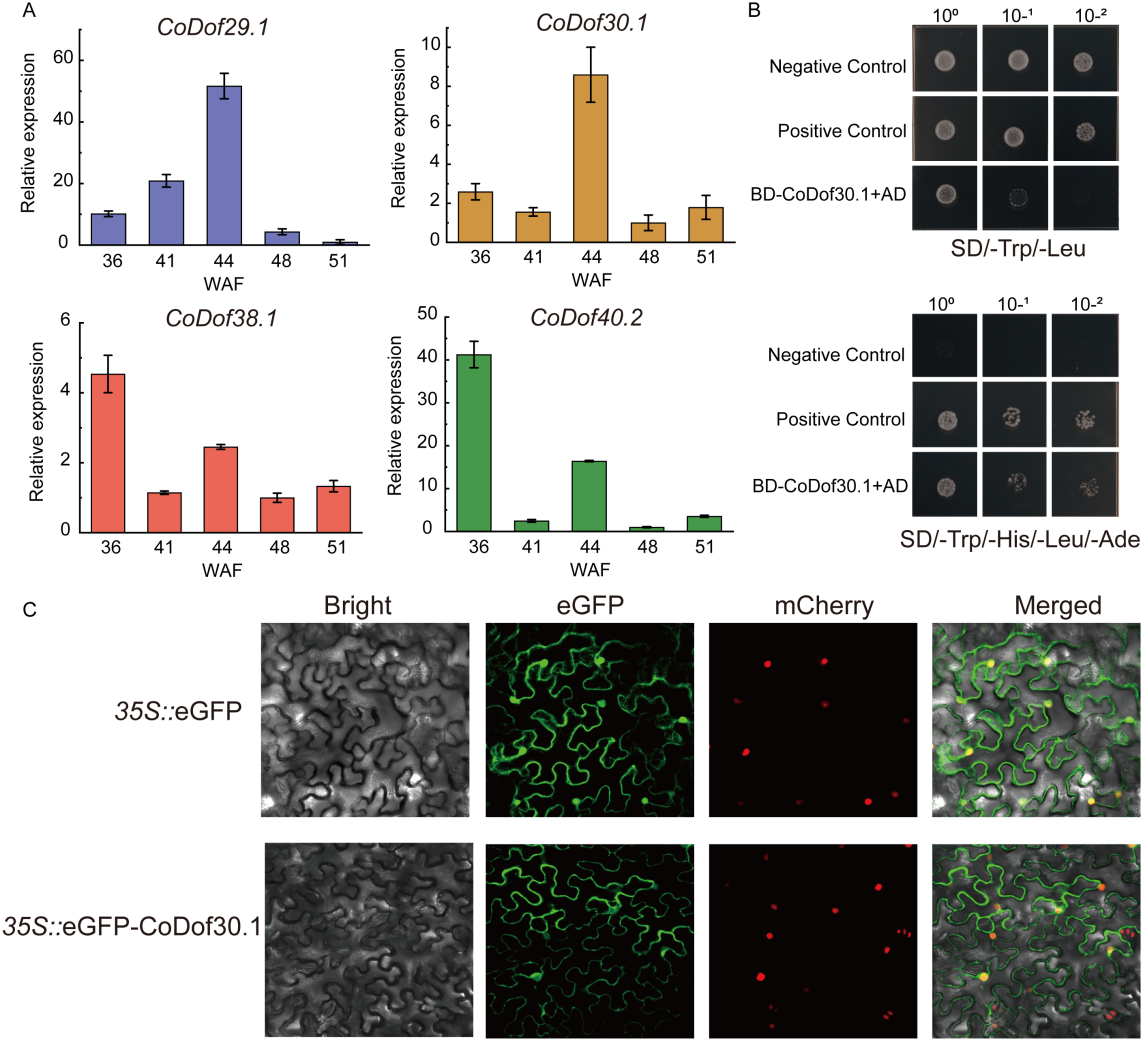
RT-qPCR analysis, transcriptional activity, and subcellular localization of CoDof30 protein. **(A)** RT-qPCR analysis of highly correlation *CoDof* genes; **(B)** Functional characterization of *CoDof30.1* transcriptional activation was performed through yeast hybrid assays. Experimental design included BD-53 + AD-T as positive controls and pGBKT7 + pGADT7 as negative controls; **(C)** Subcellular localization analysis revealed distinct patterns when comparing 35S::*CoDof30.1*-GFP fusion constructs with empty vector controls in transiently transformed tobacco leaf epidermis. Fluorescence microscopic examination (20 μm scale bar) demonstrated nuclear-specific accumulation of the fusion protein, contrasting with the diffuse cytoplasmic-nuclear distribution observed in GFP-only controls.

To validate the expression patterns in seed differential stages of four candidate *CoDof* transcription factors (*CoDof29.1*, *CoDof38.1*, *CoDof40.2*, and *CoDof30.1*), quantitative reverse transcription PCR (RT-qPCR) was performed. Comparative analysis revealed strong concordance between the RT-qPCR results and the RNA-seq data, confirming consistent expression trends across both methodologies. Among them, *CoDof29.1* and *CoDof30.1* were highly expressed in the middle stage of seed development (44 weeks seed), while *CoDof38.1* and *CoDof40.2* were specifically expression in the early stage of seed development (36 and 41 weeks seed). This indicated that *CoDof*s may exert important regulatory effects in the fatty acids and lipid pathway during the stages of seed development in COL-tetra (**Figure 8A**). To investigate the transcriptional activation potential of *CoDof30.1*, we conducted yeast hybrid assays using Y2H Gold strains carrying BD-CoDof30.1 fusion constructs. Growth patterns comparable to positive controls were observed on selective SD/-Trp/-Ade/-His/-Leu media, demonstrating significant transcriptional activation capacity (**Figure 8B**). Subcellular distribution analysis was performed through transient expression of 35S::*CoDof30*-eGFP constructs in tobacco leaf epidermal cells. Confocal microscopy revealed distinct localization patterns: while control 35S::eGFP constructs showed ubiquitous cytoplasmic and nuclear fluorescence, and the CoDof30 fusion protein exhibited nuclear and membrane accumulation (**Figure 8C**). These findings confirm nuclear and membrane localization and functional role as a transcription activator of *CoDof30*, which considered as a key candidate *Dof* transcription factor involved in fatty acid and lipid process.

## 3 Discussion

Fatty acid and lipid biosynthesis constitute core biochemical processes enabling plants to dynamically allocate metabolic resources and adapt to environmental fluctuations. These pathways serve as central hubs in plant metabolism, orchestrating energy storage, developmental regulation, and stress adaptation through tightly coordinated enzymatic cascades (Afifi et al., 2025). These pathways involve a series of interconnected enzymatic reactions, including elongation, isomerization, and conversion of fatty acids, ultimately resulting in the production of lipids essential for plant growth, development, and defense mechanisms (Baud and Lepiniec, 2010). Emerging as pivotal metabolic regulators, *DNA-binding with one finger* (*Dof*) transcription factors represent a phylogenetically conserved zinc finger subclass unique to plants, garnering considerable research interest for their master regulatory capacity in fine-tuning lipid homeostasis and fatty acid metabolic networks (Ibáñez-Salazar et al., 2014). Notably, promoter regions of numerous lipid metabolism-related genes contain dense concentrations of Dof-binding motifs, where these transcription factors function as nuclear transcriptional activators through direct DNA interaction. This characteristic finding provides novel insights into the potential involvement of *Dof* genes in lipid biosynthesis regulation. The soybean-derived Dof-type transcription factors *GmDof4* and *GmDof11* significantly increased total fatty acid and oil content in transgenic *Arabidopsis* seeds (Wang et al., 2007). Mechanistic analyses reveal that *Dof* transcription factors mediate lipid biogenesis through transcriptional activation of core lipidogenic enzymes, particularly by enhancing the expression of genes encoding fatty acid elongases, desaturases, and acyltransferases biosynthesis.

Based on the genome of COL-tetra, this study identified 40 *CoDof* family members and investigated their fundamental characteristics and expression patterns through comparative genomics. The number of *CoDof* genes varies significantly across plant species, primarily due to differences in genome size, complexity, and evolutionary history (Wang et al., 2022). Notably, a single haplotype subgenome of tetraploid *C. oleifera* harbors 40 *CoDof* genes, surpassing the counts in *Arabidopsis thaliana*, *Triticum aestivum*, and *Oryza sativa*, while being comparable to that of *Zea mays* (Jiang et al., 2012). However, the number of 45 *ColDof* members identified in previous studies is larger than that in our study (Fu et al., 2024). This discrepancy in transcription factor counts stems is mainly due to the fact that their reference genome is a wild progenitor *C.oleifera* genome (Lin et al., 2022), which taxonomically belongs to an unphased haplotype assembly species compared with COL-tetra. This assembly approach risks misannotating divergent alleles as separate genes, whereas the phased tetraploid genome properly resolves allelic variants across homologous chromosomes, inherently leading to gene quantification variances (Zhang et al., 2024).

All identified *CoDof* genes and their allelic counterparts encode proteins containing a conserved single C_2_-C_2_ zinc finger domain. Sequence alignment revealed that the zinc finger motifs in *CoDof* transcription factors exhibit high conservation across critical amino acid residues, suggesting evolutionary preservation of their DNA-binding functionality. The *Dof* transcription factor family in *Arabidopsis thaliana* is divided into nine subfamilies based on phylogenetic analysis (Lijavetzky et al., 2003). All subfamilies contain *CoDof* gene members, which are widely involved in plant growth and development regulation. In the D1 subfamily, overexpression of *Arabidopsis CDF3* gene delays flowering and significantly enhances plant tolerance to low temperature and drought stresses (Corrales et al., 2017). In rice, overexpression of *OsDof3* and *OsDof11* promotes early flowering under long-day conditions; however, *OsDof3* overexpression delays flowering under short-day conditions, while *OsDof11* overexpression has no significant effect on flowering time (Li et al., 2009). These findings suggest that D1 subfamily members such as *CoDof3.1*, *CoDof28.1*, and *CoDof26.1* may play roles in regulating plant flowering time. Besides, subfamily members exhibit functional and structural conservation (Moreno-Risueno et al., 2007), including ectopic expression of A subfamily *OBP4* in *Arabidopsis* inhibits cell elongation, leading to dwarfism (Rymen et al., 2017). In COL-tetra, *CoDof* genes in A subclade share conserved motifs and gene structures, implying their potential roles in regulating growth and development. Additionally, *Arabidopsis AtDof2.3* in the D2 subfamily shows a 69.46-fold upregulation under PEG-induced drought stress, and its overexpression significantly increases root length and activities of peroxidase and superoxide dismutase in drought-treated plants. Overexpression of D3 subfamily *AtDof5.8* confers enhanced resistance to drought and salt stresses in transgenic *Arabidopsis* (He et al., 2015). This functional plasticity inherent in Dof proteins enables their sophisticated coordination of plant metabolic networks, spanning photosynthetic regulation, secondary metabolite production, and stress-responsive transcriptional programs.

Moreover, cis-elements like ABRE, TC-rich repeats, TCA-element, and ARE were implicated in abscisic acid responsiveness or anaerobic induction (Wang et al., 2020). Hormone-related cis-elements, including ERE, CGTCA-motif, TGACG-motif, and GARE-motif, were also present in the *CoDof* promoter region. The diverse domains, structures, and cis-elements within the promoter region collectively contribute to the varied functions exhibited by the *CoDof* family of transcription factors. Analysis of expression abundance in 40 pairs of *CoDof* alleles revealed that in certain cases, there exists imbalanced expression between alleles, which may be regulated by various external and internal factors. For example, *CoDof38.1, CoDof38.3, CoDof38.5* are highly expressed in tender leaves and leaf buds, but *CoDof38.2, CoDof38.6 are only* highly expressed in late development stage of seed.

Diacylglycerol acyltransferase (DGAT) represents the final and rate-limiting enzyme in the acyl-CoA-dependent Kennedy pathway for triacylglycerol (TAG) biosynthesis. The WRI1 transcription factor directly interacts with key fatty acid biosynthesis genes such as *KASI* and *BCCP* to regulate fatty acid synthesis and oil accumulation. Studies in *Arabidopsis* have revealed a synergistic effect between *WRI1* and *DGAT1*, where co-overexpression of both genes leads to significantly higher seed oil content compared to single-gene overexpression (Ćurko et al., 2014). In COL-tetra, combined actions of *SAD/FADs*, and *CoDGAT1* have been identified as the molecular basis for high oleic acid content in seed oils. In this study, building upon the established expression profiles and regulatory relationships between fatty acid biosynthesis genes and *CoDof* transcription factors (identified through Pearson correlation analysis), we specifically targeted genes demonstrating significant positive associations (r > 0.5, *p* < 0.01) with *SAD* and *DGAT*. Besides, we postulated *CoDof30* as a key regulatory gene mediating fatty acid/lipid biosynthesis in COL-tetra seeds. Experimental validation revealed its dual nucleo-cytoplasmic membrane localization coupled with robust transactivation capacity, which may be involved in fatty acid/lipid biosynthesis.

In conclusion, this study systematically and firstly identified 40 CoDof genes exhibiting allelic diversity (116 alleles) in the COL-tetra. Integrated multi-omics analysis, incorporating physiological metabolite tracking, and acyl-lipid metabolic machinery correlation networks, revealing stage-specific expression divergence during seed maturation phases. The *CoDof30.1* as a nucleus-localized transcriptional regulator showing peak activity during the lipid hyper-accumulation phase. These findings establish a molecular framework for elucidating the functional divergence of COL-tetra *CoDof* paralogs in modulating fatty acid/lipid metabolic pathways, particularly those governing oleochemical biosynthesis.

## 4 Materials and methods

### 4.1 Identification of *CoDof* transcription factors in the COL-tetra

To elucidate the DNA binding characteristics of the one finger family (Dof) of transcription factors, the hidden Markov model (HMM) profile of the Dof domain, a zinc finger DNA-binding domain (PF02701), was utilized, sourced from the Pfam database (El-Gebali et al., 2019). The reference genomes of the COL-tetra served as the foundational basis for this investigation (Zhang et al., 2024). The presence of conserved domains within the Dof family members was validated using HMMER SEARCH 3.0 with a cutoff E-value of ≤ 0.01, in conjunction with the SMART database (Finn et al., 2011a). Theoretical isoelectric point (pI) and molecular weight (Mw) values were computed using the ExPaSy online tool (https://web.expasy.org/compute_pi/) (Finn et al., 2011b). For protein modeling, the SWISS-MODEL online tool (https://swissmodel.expasy.org/) was employed (Gasteiger et al., 2003), while the putative subcellular localization of genes was predicted using the Softberry online software (http://www.softberry.com).

### 4.2 Conserved motifs, gene structures, and phylogenetic analysis of *CoDof* members

Conserved motifs were identified utilizing the MEME online tool (Version 5.1.0, National Institutes of Health, Bethesda, MD, USA), and the aligned domains were visualized with DNAMAN (Version 8.0.8, Lynnon Biosoft, San Ramon, CA, USA). The gene structures of *CoDof*s were predicted using the Gene Structure Display Server (Kumar et al., 2018). An unrooted phylogenetic tree for CoDofs and AtDofs proteins was constructed using Maximum Likelihood (ML) method, with bootstrap testing conducted over 1000 replicates, using MEGA11 (https://www.megasoftware.net/home) (Chen et al., 2023).

### 4.3 Cis-elements analysis of *CoDofs*

The promoter regions, defined as the 2000 base pair sequences upstream of the coding DNA sequences (CDS), were examined for cis-regulatory elements utilizing the PlantCARE web tool (http://bioinformatics.psb.ugent.be/webtools/plantcare/html/) (Lescot et al., 2002).

### 4.4 Materials and RNA-seq analysis

The seed materials for this research were sourced from 8-year-old Camellia oleifera trees planted in the experimental zone of Central South University of Forestry and Technology, located in Jiayi Town, Pingjiang County, Hunan Province, China (113°51’1” N, 28°38’21’’ E). This area experiences a subtropical monsoon climate with an annual frost-free period of approximately 275 days. The average yearly temperature is 16.8°C, and the average annual rainfall is 1450.8 mm. Seed samples were gathered at five different developmental periods: 36, 41, 44, 48, and 51 weeks after flowering (WAF), and were stored at -80°C until needed. The raw RNA-seq data (GWHERBT00000000) used in the study were sourced from the National Genomics Data Center (https://ngdc.cncb.ac.cn/) (Zhang et al., 2024). GO enrichment analyses were conducted using the Plant Transcriptional Regulatory Map (Jin et al., 2017), with a significance level set at a corrected p-value < 0.05. KEGG analyses were performed using KOBAS-i: intelligence online tools (http://bioinfo.org/kobaskobas3/?t=1) and visualized with R (Bu et al., 2021).

### 4.5 COL-tetra seed oil analysis

*C. oleifera* seeds were oven-dried at 60°C to a constant weight, ground into powder, and 0.1 g was placed in a 15 mL centrifuge tube. After adding 4 mL n-heptane and 100 μL of a C12:0 internal standard, the mixture was vortexed for 15 seconds and rested for 30 seconds, repeating this cycle three times. Then, 200 μL of 2 mol/L potassium hydroxide methanol solution was added and vortexed until clear. After adding 1 g anhydrous sodium sulfate and vortexing again, the supernatant was filtered through a 0.45 μm filter and prepared for gas chromatography analysis.

The gas chromatography (GC) analysis was conducted utilizing a Shimadzu GC2014 system (Kyoto, Japan), which was equipped with a DB-WAX column (30 m; Agilent Technologies, Palo Alto, CA, USA). Nitrogen was employed as the carrier gas at a split ratio of 20:1. Detection was achieved using a hydrogen flame ionization detector, maintained at a temperature of 230°C. The column temperature program was configured as follows: an initial temperature of 100°C was maintained for 5 minutes, followed by an increase to 220°C at a rate of 10°C per minute, with a final hold at 220°C for 15 minutes. The oil content was calculated using the formula: Oil content = [(4/percentage of internal standard (C12:0) - 4)/weight of powder] × 100%. For the determination of oil content, three biological replicates were performed for each developmental period.

### 4.6 Quantitative reverse transcription PCR analysis

Total RNA was extracted using the TransZol kit (TransGen Biotech, Inc., Beijing, China), and complementary DNA (cDNA) synthesis was conducted with the HiScript II QRT SuperMix for qPCR (+gDNA wiper) kit from Vazyme (Piscataway, NJ, USA). DNase was used DNase I, RNase-free from Vazyme (Piscataway, NJ, USA). RT-qPCR experiments were performed utilizing the LightCycler 96 Real-Time PCR System from Roche (Basel, Switzerland). A reaction volume of 50 μL was prepared, with each sample subjected to three biological replicates and three technical replicates. The HiScript II Q RT SuperMix for qPCR (+gDNA wiper) kit from Vazyme was used for reverse transcription. Relative expression levels were calculated using the 2^−ΔΔCt^ method, with *CoEF1α* serving as the internal reference gene for normalization. Expression levels were normalized to the sample exhibiting the lowest expression. Details of the primers for *CoDofs* and the *CoEF1α* gene are provided in **Supplementary Table S3**. Data are presented as mean ± standard deviations (SD), with statistical significance determined by Student’s t-test (* *p* < 0.05, ** *p* < 0.01, *** *p* < 0.001).

### 4.7 Pearson correlation coefficient and transcriptional activity analysis

The Pearson correlation coefficient (r) was employed to assess the correlation between fatty acids and lipid biosynthesis genes and *CoDof* transcription factors. A higher absolute value of r indicates a stronger correlation, with values exceeding 0.5 depicted in **Figure 6A**. The complete coding sequence (CDS) of *CoDof30.1* was cloned into the pGBDKT7 vector. Subsequently, the constructs pGBDKT7- *CoDof30.1*, pGBDKT7-53 (positive control), and pGBDKT7-lam (negative control) were introduced into the Y2HGold yeast strain. The transformed yeast cells were cultured on three types of media: SD/-Trp and SD/-Trp/-His/-Ade plates. Details of the primers used are provided in **Supplementary Table S3**.

### 4.8 Subcellular localization assay of *CoDof30.1*

The complete coding sequence (CDS) of *CoDof30.1*, excluding the stop codon, was inserted into the pSUPER1300 vector and subsequently fused with the green fluorescent protein (GFP) to create a CoDof30.1-GFP fusion protein. The empty pSUPER1300 vector was utilized as the control. These constructions were introduced into Agrobacterium tumefaciens strain GV3101. Co-transformation of the GFP-fusion constructs and the nuclear localization marker (NF-YA4-mCherry) was conducted in *Nicotiana benthamiana* leaves. After 48 hours post-infiltration, fluorescence signals were detected at excitation/emission wavelengths of 488/510 nm for GFP and 552/610 nm for mCherry using a confocal laser scanning microscope (Leica SP8, Heidelberg, Germany). Primer details are provided in **Supplementary Table S3**.

## Data Availability

The datasets presented in this study can be found in online repositories. The names of the repository/repositories and accession number(s) can be found below: https://ngdc.cncb.ac.cn/, GWHERBT00000000.

## References

[B1] Afifi E. H. John Martin J. J. Wang Q. Li X. Liu X. Zhou L.. (2025). Fatty acid and lipid metabolism in oil palm: from biochemistry to molecular mechanisms. Int. J. Mol. Sci. 26, 2531. doi: 10.3390/ijms26062531 40141173 PMC11942028

[B2] Austin M. B. Noel J. P. (2003). The chalcone synthase superfamily of type III polyketide synthases. Nat. Prod Rep. 20, 79–110. doi: 10.1039/b100917f 12636085

[B3] Baud S. Lepiniec L. (2010). Physiological and developmental regulation of seed oil production. Prog. Lipid Res. 49, 235–249. doi: 10.1016/j.plipres.2010.01.001 20102727

[B4] Bu D. Luo H. Huo P. Wang Z. Zhang S. He Z.. (2021). KOBAS-i: intelligent prioritization and exploratory visualization of biological functions for gene enrichment analysis. Nucleic Acids Res. 49, W317–W325. doi: 10.1093/nar/gkab447 34086934 PMC8265193

[B5] Chen C. Wu Y. Li J. Wang X. Zeng Z. Xu J.. (2023). TBtools-II: A “one for all, all for one” bioinformatics platform for biological big-data mining. Mol. Plant 16, 1733–1742. doi: 10.1016/j.molp.2023.09.010 37740491

[B6] Corrales A. R. Carrillo L. Lasierra P. Nebauer S. G. Dominguez-Figueroa J. Renau-Morata B.. (2017). Multifaceted role of cycling DOF factor 3 (CDF3) in the regulation of flowering time and abiotic stress responses in Arabidopsis. Plant Cell Environ. 40, 748–764. doi: 10.1111/pce.12894 28044345

[B7] Ćurko N. Kovačević Ganić K. Gracin L. Ðapić M. Jourdes M. Teissedre P. L. (2014). Characterization of seed and skin polyphenolic extracts of two red grape cultivars grown in Croatia and their sensory perception in a wine model medium. Food Chem. 145, 15–22. doi: 10.1016/j.foodchem.2013.07.131 24128443

[B8] El-Gebali S. Mistry J. Bateman A. Eddy S. R. Luciani A. Potter S. C.. (2019). The Pfam protein families database in 2019. Nucleic Acids Res. 47, D427–D432. doi: 10.1093/nar/gky995 30357350 PMC6324024

[B9] Finn R. D. Clements J. Eddy S. R. (2011). HMMER web server: interactive sequence similarity searching. Nucleic Acids Res. 39, W29–W37. doi: 10.1093/nar/gkr367 21593126 PMC3125773

[B10] Fu C. Xiao Y. Jiang N. Yang Y. (2024). Genome-wide identification and molecular evolution of Dof gene family in Camellia oleifera. BMC Genomics 25, 702. doi: 10.1186/s12864-024-10622-6 39026173 PMC11264790

[B11] Gasteiger E. Gattiker A. Hoogland C. Ivanyi I. Appel R. D. Bairoch A. (2003). ExPASy: The proteomics server for in-depth protein knowledge and analysis. Nucleic Acids Res. 31, 3784–3788. doi: 10.1093/nar/gkg563 12824418 PMC168970

[B12] He L. Su C. Wang Y. Wei Z. (2015). ATDOF5.8 protein is the upstream regulator of ANAC069 and is responsive to abiotic stress. Biochimie 110, 17–24. doi: 10.1016/j.biochi.2014.12.017 25572919

[B13] Ibáñez-Salazar A. Rosales-Mendoza S. Rocha-Uribe A. Ramírez-Alonso J. I. Lara-Hernández I. Hernández-Torres A.. (2014). Over-expression of Dof-type transcription factor increases lipid production in Chlamydomonas reinhardtii. J. Biotechnol. 184, 27–38. doi: 10.1016/j.jbiotec.2014.05.003 24844864

[B14] Jiang Y. Zeng B. Zhao H. Zhang M. Xie S. Lai J. (2012). Genome-wide transcription factor gene prediction and their expressional tissue-specificities in maize. J. Integr. Plant Biol. 54, 616–630. doi: 10.1111/j.1744-7909.2012.01149.x 22862992

[B15] Jin J. Tian F. Yang D. C. Meng Y. Q. Kong L. Luo J.. (2017). PlantTFDB 4.0: toward a central hub for transcription factors and regulatory interactions in plants. Nucleic Acids Res. 45, 1040–1045. doi: 10.1093/nar/gkw982 PMC5210657 27924042

[B16] Kim H. S. Kim S. J. Abbasi N. Bressan R. A. Yun D. J. Yoo S. D.. (2010). The DOF transcription factor Dof5.1 influences leaf axial patterning by promoting Revoluta transcription in Arabidopsis. Plant J. 64, 524–535. doi: 10.1111/j.1365-313X.2010.04346.x 20807212

[B17] Kumar S. Stecher G. Li M. Knyaz C. Tamura K. (2018). MEGA X: molecular evolutionary genetics analysis across computing platforms. Mol. Biol. Evol. 35, 1547–1549. doi: 10.1093/molbev/msy096 29722887 PMC5967553

[B18] Lescot M. Déhais P. Thijs G. Marchal K. Moreau Y. Van de Peer Y.. (2002). PlantCARE, a database of plant cis-acting regulatory elements and a portal to tools for in silico analysis of promoter sequences. Nucleic Acids Res. 30, 325–327. doi: 10.1093/nar/30.1.325 11752327 PMC99092

[B19] Li D. Yang C. Li X. Gan Q. Zhao X. Zhu L. (2009). Functional characterization of rice OsDof12. Planta. 229, 1159–1169. doi: 10.1007/s00425-009-0893-7 19198875

[B20] Lijavetzky D. Carbonero P. Vicente-Carbajosa J. (2003). Genome-wide comparative phylogenetic analysis of the rice and Arabidopsis Dof gene families. BMC Evol. Biol. 3, 17. doi: 10.1186/1471-2148-3-17 12877745 PMC184357

[B21] Lin P. Wang K. Wang Y. Hu Z. Yan C. Huang H.. (2022). The genome of oil-Camellia and population genomics analysis provide insights into seed oil domestication. Genome Biol. 23, 14. doi: 10.1186/s13059-021-02599-2 35012630 PMC8744323

[B22] Moreno-Risueno M. A. Martínez M. Vicente-Carbajosa J. Carbonero P. (2007). The family of DOF transcription factors: from green unicellular algae to vascular plants. Mol. Genet. Genomics 277, 379–390. doi: 10.1007/s00438-006-0186-9 17180359

[B23] Nagaoka S. Takeuchi A. Banno A. (2021). Plant-derived peptides improving lipid and glucose metabolism. Peptides. 142, 170577. doi: 10.1016/j.peptides.2021.170577 34033874

[B24] Noguero M. Atif R. M. Ochatt S. Thompson R. D. (2013). The role of the DNA-binding One Zinc Finger (DOF) transcription factor family in plants. Plant Sci. 209, 32–45. doi: 10.1016/j.plantsci.2013.03.016 23759101

[B25] Ohlrogge J. Browse J. (1995). Lipid biosynthesis. Plant Cell. 7, 957–970. doi: 10.1105/tpc.7.7.957 7640528 PMC160893

[B26] Rymen B. Kawamura A. Schäfer S. Breuer C. Iwase A. Shibata M.. (2017). ABA suppresses root hair growth via the OBP4 transcriptional regulator. Plant Physiol. 173, 1750–1762. doi: 10.1104/pp.16.01945 28167701 PMC5338652

[B27] Sharif R. Thomas P. Zalewski P. Fenech M. (2012). The role of zinc in genomic stability. Mutat. Res. 733, 111–121. doi: 10.1016/j.mrfmmm.2011.08.009 21939673

[B28] Shimano H. Sato R. (2017). SREBP-regulated lipid metabolism: convergent physiology - divergent pathophysiology. Nat. Rev. Endocrinol. 13, 710–730. doi: 10.1038/nrendo.2017.91 28849786

[B29] Skirycz A. Radziejwoski A. Busch W. Hannah M. A. Czeszejko J. Kwaśniewski M.. (2008). The DOF transcription factor OBP1 is involved in cell cycle regulation in Arabidopsis thaliana. Plant J. 56, 779–792. doi: 10.1111/j.1365-313X.2008.03641.x 18665917

[B30] Smith S. Witkowski A. Joshi A. K. (2003). Structural and functional organization of the animal fatty acid synthase. Prog. Lipid Res. 42, 289–317. doi: 10.1016/S0163-7827(02)00067-X 12689621

[B31] Wang J. Liu Y. Tang B. Dai X. Xie L. Liu F.. (2020). Genome-wide identification and capsaicinoid biosynthesis-related expression analysis of the *R2R3-MYB* gene family in *capsicum annuum* L. Front. Genet. 11, 598183. doi: 10.3389/fgene.2020.598183 33408738 PMC7779616

[B32] Wang J. Yang G. Chen Y. Dai Y. Yuan Q. Shan Q.. (2022). Genome-wide characterization and anthocyanin-related expression analysis of the *B-BOX* gene family in *capsicum annuum* L. Front. Genet. 13, 847328. doi: 10.3389/fgene.2022.847328 35295945 PMC8918674

[B33] Wang H. W. Zhang B. Hao Y. J. Huang J. Tian A. G. Liao Y.. (2007). The soybean Dof-type transcription factor genes, GmDof4 and GmDof11, enhance lipid content in the seeds of transgenic Arabidopsis plants. Plant J. 52, 716–729. doi: 10.1111/j.1365-313X.2007.03268.x 17877700

[B34] Wei P. C. Tan F. Gao X. Q. Zhang X. Q. Wang G. Q. Xu H.. (2010). Overexpression of AtDOF4.7, an Arabidopsis DOF family transcription factor, induces floral organ abscission deficiency in Arabidopsis. Plant Physiol. 153, 1031–1045. doi: 10.1104/pp.110.153247 20466844 PMC2899910

[B35] Wei Q. Wang W. Hu T. Hu H. Mao W. Zhu Q.. (2018). Genome-wide identification and characterization of *Dof* transcription factors in eggplant (*Solanum melongena* L.). PeerJ 6, e4481. doi: 10.7717/peerj.4481 29527420 PMC5844252

[B36] Wu Q. Li D. Li D. Liu X. Zhao X. Li X.. (2015). Overexpression of OsDof12 affects plant architecture in rice (*Oryza sativa* L. ). Front. Plant Sci. 6, 833. doi: 10.3389/fpls.2015.00833 26500670 PMC4597119

[B37] Xia Y. Yu K. Navarre D. Seebold K. Kachroo A. Kachroo P. (2010). The glabra1 mutation affects cuticle formation and plant responses to microbes. Plant Physiol. 154, 833–846. doi: 10.1104/pp.110.161646 20699396 PMC2949009

[B38] Yanagisawa S. (1997). Dof DNA-binding domains of plant transcription factors contribute to multiple protein-protein interactions. Eur. J. Biochem. 250, 403–410. doi: 10.1111/j.1432-1033.1997.0403a.x 9428691

[B39] Yang D. Wang R. Lai H. He Y. Chen Y. Xun C.. (2024). Comparative transcriptomic and lipidomic analysis of fatty acid accumulation in three *camellia oleifera* varieties during seed maturing. J. Agric. Food Chem. 72, 18257–18270. doi: 10.1021/acs.jafc.4c03614 39084609 PMC11328181

[B40] Yoneshiro T. Wang Q. Tajima K. Matsushita M. Maki H. Igarashi K.. (2019). BCAA catabolism in brown fat controls energy homeostasis through SLC25A44. Nature. 572, 614–619. doi: 10.1038/s41586-019-1503-x 31435015 PMC6715529

[B41] Zhang C. Dong T. Yu J. Hong H. Liu S. Guo F.. (2023). Genome-wide survey and expression analysis of Dof transcription factor family in sweetpotato shed light on their promising functions in stress tolerance. Front. Plant Sci. 14, 1140727. doi: 10.3389/fpls.2023.1140727 36895872 PMC9989284

[B42] Zhang L. Shi Y. Gong W. Zhao G. Xiao S. Lin H.. (2024). The tetraploid Camellia oleifera genome provides insights into evolution, agronomic traits, and genetic architecture of oil Camellia plants. Cell Rep. 43, 115032. doi: 10.1016/j.celrep.2024.115032 39576729

