# Supplementary figures and images for: Identification and expression profiling of the CoDof genes involved in fatty acid/lipid biosynthesis of tetraploid Camellia oleifera
Source: Front Plant Sci. 2025 Jun 9;16:1599849. doi: 10.3389/fpls.2025.1599849 (PMC12183095; doi:10.3389/fpls.2025.1599849)

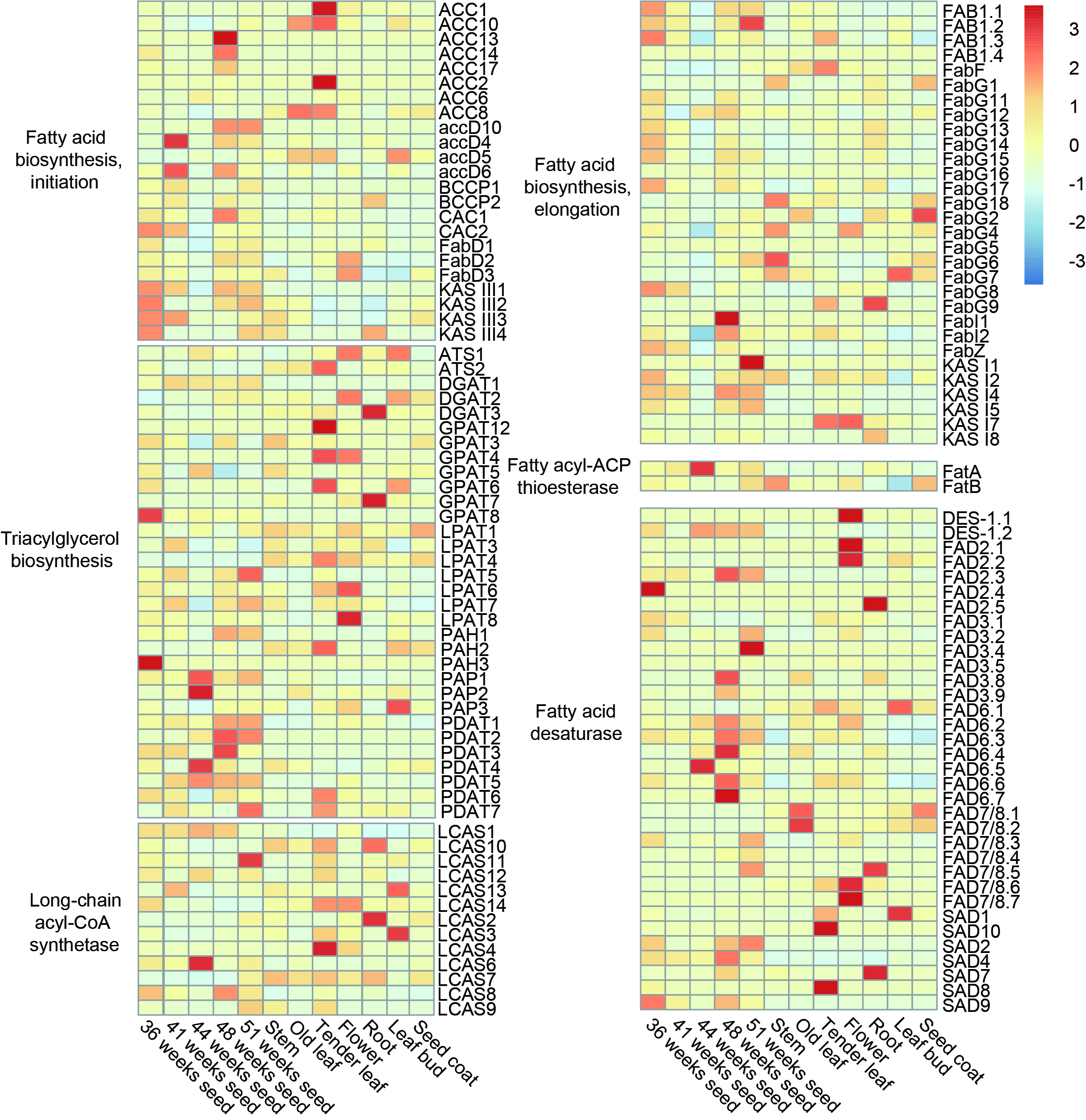

Supplement: Supplementary file 1 [file Image1.jpeg]
