# Supplementary material for: Identification and expression profiling of the CoDof genes involved in fatty acid/lipid biosynthesis of tetraploid Camellia oleifera
Source: Front Plant Sci. 2025 Jun 9;16:1599849. doi: 10.3389/fpls.2025.1599849 (PMC12183095; doi:10.3389/fpls.2025.1599849)
Supplement: Supplementary file 2 [file Table1.docx]

Table S1. Basic information of *CoDof* family members

| gene id | CoDof_ID | Number of Amino Acid | Molecular Weight | Theoretical pI | Instability Index | Aliphatic Index | Grand Average of Hydropathicity |
| --- | --- | --- | --- | --- | --- | --- | --- |
| YC.01G0004250-1A | CoDof1.1 | 324 | 34282.28 | 9.23 | 52.87 | 57.78 | -0.514 |
| YC.01G0004250-2B | CoDof1.2 | 345 | 36679.89 | 9.06 | 54.07 | 61.59 | -0.477 |
| YC.01G0004250-3C | CoDof1.3 | 346 | 36487.58 | 9.2 | 53.73 | 57.77 | -0.545 |
| YC.01G0013970-1A | CoDof2.1 | 223 | 23414.95 | 7.56 | 52.57 | 50.22 | -0.512 |
| YC.01G0013970-2B | CoDof2.2 | 346 | 36616.74 | 9.32 | 53.33 | 57.77 | -0.558 |
| YC.01G0013970-3C | CoDof2.3 | 324 | 34236.21 | 9.23 | 54.17 | 56.88 | -0.531 |
| YC.01G0017380-1A | CoDof3.1 | 224 | 23498.02 | 7.58 | 52.07 | 51.29 | -0.505 |
| YC.01G0017380-2B | CoDof3.2 | 318 | 35573.8 | 6.14 | 47.32 | 52.39 | -0.714 |
| YC.01G0017380-3C | CoDof3.3 | 244 | 26931.9 | 4.89 | 49.3 | 63.11 | -0.511 |
| YC.01G0017380-4D | CoDof3.4 | 309 | 34626.63 | 6.14 | 46.14 | 50.13 | -0.776 |
| YC.01G0041710-1D | CoDof4 | 275 | 30796.31 | 7.25 | 47.28 | 50.69 | -0.845 |
| YC.01G0042290-1D | CoDof5 | 270 | 30115.67 | 7.63 | 46.81 | 51.63 | -0.794 |
| YC.02G0008890-1A | CoDof6.1 | 318 | 35583.84 | 6.14 | 47.32 | 52.39 | -0.716 |
| YC.02G0008890-2B | CoDof6.2 | 324 | 34218.18 | 9.23 | 52.73 | 58.09 | -0.523 |
| YC.02G0008890-3C | CoDof6.3 | 224 | 23482.02 | 7.58 | 51.69 | 51.74 | -0.494 |
| YC.02G0016270-1A | CoDof7.1 | 244 | 26945.93 | 4.89 | 49.3 | 63.52 | -0.513 |
| YC.02G0016270-2B | CoDof7.2 | 84 | 9251.21 | 8.63 | 68.55 | 129.88 | 0.351 |
| YC.02G0016270-3C | CoDof7.3 | 270 | 30155.7 | 7.64 | 47.09 | 51.63 | -0.8 |
| YC.02G0016270-4D | CoDof7.4 | 244 | 26888.87 | 4.81 | 51.42 | 64.71 | -0.508 |
| YC.02G0016930-1A | CoDof8.1 | 351 | 36960.22 | 8.79 | 53.62 | 58.58 | -0.481 |
| YC.02G0016930-2B | CoDof8.2 | 133 | 15046.75 | 9.6 | 57.06 | 38.2 | -0.995 |
| YC.02G0016930-3D | CoDof8.3 | 307 | 34406.46 | 6.1 | 46.26 | 49.87 | -0.748 |
| YC.03G0035170-1C | CoDof9 | 281 | 30738.19 | 9.26 | 43.09 | 55.84 | -0.664 |
| YC.04G0003700-1A | CoDof10.1 | 267 | 29128.46 | 9.48 | 44.26 | 58.76 | -0.654 |
| YC.04G0003700-2B | CoDof10.2 | 281 | 30704.17 | 9.26 | 43.09 | 57.22 | -0.66 |
| YC.04G0003700-3D | CoDof10.3 | 306 | 33145.24 | 9.03 | 56.23 | 58.69 | -0.481 |
| YC.04G0007230-1A | CoDof11.1 | 306 | 33227.34 | 8.92 | 55.8 | 59.64 | -0.477 |
| YC.04G0007230-2B | CoDof11.2 | 267 | 29158.48 | 9.48 | 44.26 | 58.39 | -0.664 |
| YC.04G0007230-3C | CoDof11.3 | 307 | 34491.57 | 6.21 | 46.29 | 49.87 | -0.761 |
| YC.04G0011140-1A | CoDof12.1 | 307 | 34307.32 | 5.98 | 45.88 | 49.87 | -0.734 |
| YC.04G0011140-2B | CoDof12.2 | 307 | 34259.28 | 5.98 | 45.6 | 50.81 | -0.73 |
| YC.04G0011140-3D | CoDof12.3 | 260 | 28679.77 | 9.07 | 57.91 | 45.08 | -0.857 |
| YC.04G0019150-1A | CoDof13.1 | 105 | 11477.69 | 9.14 | 63.39 | 48.29 | -0.788 |
| YC.04G0019150-2B | CoDof13.2 | 251 | 27856.71 | 9.64 | 67.19 | 44.7 | -1.077 |
| YC.04G0019150-3D | CoDof13.3 | 161 | 18141.5 | 8.94 | 43.88 | 49.69 | -0.684 |
| YC.04G0023440-1A | CoDof14.1 | 255 | 28313.3 | 9.51 | 63.1 | 44 | -1.022 |
| YC.04G0023440-2B | CoDof14.2 | 328 | 36339.22 | 8.63 | 54.01 | 58.51 | -0.746 |
| YC.04G0023440-1T | CoDof14.3 | 287 | 31697.96 | 6.75 | 55.31 | 55.05 | -0.707 |
| YC.04G0023440-3C | CoDof14.4 | 178 | 19983.37 | 9.22 | 55.44 | 47.13 | -0.823 |
| YC.04G0023440-4D | CoDof14.5 | 260 | 29249.03 | 9.36 | 51.34 | 61.88 | -0.553 |
| YC.04G0033500-1B | CoDof15.1 | 287 | 31731.04 | 7.07 | 55.05 | 55.05 | -0.712 |
| YC.04G0033500-2C | CoDof15.2 | 328 | 36385.29 | 8.63 | 52.58 | 59.39 | -0.738 |
| YC.04G0033500-3D | CoDof15.3 | 255 | 28267.22 | 9.57 | 63.1 | 44 | -1.034 |
| YC.05G0039460-1D | CoDof16 | 328 | 36415.27 | 8.42 | 53.67 | 58.51 | -0.761 |
| YC.06G0013490-1A | CoDof17.1 | 310 | 34516.6 | 9.26 | 72.86 | 34.68 | -1.216 |
| YC.06G0013490-2B | CoDof17.2 | 373 | 41569.54 | 8.04 | 59.14 | 71.85 | -0.542 |
| YC.06G0013490-3C | CoDof17.3 | 309 | 34391.51 | 9.26 | 73.96 | 36.05 | -1.195 |
| YC.06G0013490-4D | CoDof17.4 | 161 | 18111.47 | 8.94 | 43.88 | 50.31 | -0.669 |
| YC.06G0014510-1A | CoDof18.1 | 309 | 34365.47 | 9.26 | 73.33 | 36.38 | -1.184 |
| YC.06G0014510-2B | CoDof18.2 | 373 | 41652.62 | 8.08 | 59.2 | 71.85 | -0.551 |
| YC.06G0021260-1A | CoDof19.1 | 260 | 28328.33 | 8.18 | 58.3 | 48.04 | -0.72 |
| YC.06G0021260-2B | CoDof19.2 | 224 | 24466.03 | 8.51 | 49.71 | 43.21 | -0.717 |
| YC.06G0021260-3C | CoDof19.3 | 260 | 28273.29 | 8.18 | 58.07 | 49.19 | -0.688 |
| YC.06G0021260-4D | CoDof19.4 | 260 | 28248.15 | 7.61 | 57.72 | 46.58 | -0.732 |
| YC.07G0040590-1D | CoDof20 | 287 | 31726.02 | 6.75 | 55.31 | 55.05 | -0.707 |
| YC.08G0004080-1A | CoDof21.1 | 177 | 19855.24 | 9.22 | 54.61 | 47.4 | -0.808 |
| YC.08G0004080-2B | CoDof21.2 | 373 | 41625.59 | 8.08 | 60.37 | 71.85 | -0.543 |
| YC.08G0004080-3C | CoDof21.3 | 161 | 18113.45 | 8.94 | 45.07 | 49.13 | -0.686 |
| YC.08G0004080-4D | CoDof21.4 | 256 | 28484.55 | 9.61 | 61.08 | 46.88 | -0.97 |
| YC.08G0004730-1A | CoDof22.1 | 307 | 34132.24 | 9.26 | 73.12 | 37.56 | -1.183 |
| YC.08G0004730-2B | CoDof22.2 | 247 | 27868.06 | 4.79 | 55.14 | 62.75 | -0.639 |
| YC.08G0004730-3C | CoDof22.3 | 247 | 27798.95 | 4.72 | 54.15 | 62.75 | -0.624 |
| YC.08G0004730-4D | CoDof22.4 | 242 | 25458.94 | 6.21 | 65.84 | 47.56 | -0.664 |
| YC.08G0004760-1A | CoDof23.1 | 237 | 25295.85 | 6.3 | 62.08 | 49.79 | -0.662 |
| YC.08G0004760-2C | CoDof23.2 | 343 | 35822.78 | 9.3 | 61.98 | 51.22 | -0.586 |
| YC.08G0004760-3D | CoDof23.3 | 343 | 35870.84 | 9.3 | 63.1 | 50.09 | -0.595 |
| YC.08G0013820-1A | CoDof24.1. | 343 | 35942.99 | 9.3 | 61.15 | 50.93 | -0.57 |
| YC.08G0013820-2B | CoDof24.2. | 343 | 35870.84 | 9.3 | 61.51 | 49.8 | -0.601 |
| YC.08G0013820-3C | CoDof24.3. | 317 | 33912.48 | 9.15 | 61.98 | 48.93 | -0.68 |
| YC.08G0013820-4D | CoDof24.4. | 316 | 33856.41 | 9.03 | 61.75 | 49.08 | -0.681 |
| YC.08G0013830-1A | CoDof25.1 | 316 | 33938.5 | 9.21 | 61.09 | 49.08 | -0.691 |
| YC.08G0013830-2B | CoDof25.2 | 133 | 15265.97 | 9.54 | 56.69 | 38.2 | -1.046 |
| YC.08G0013830-3D | CoDof25.3 | 316 | 33855.43 | 9.15 | 61.75 | 49.08 | -0.681 |
| YC.08G0021750-1B | CoDof26.1 | 268 | 29622.07 | 8.38 | 61.12 | 57.05 | -0.708 |
| YC.08G0021750-2C | CoDof26.2 | 268 | 29640.11 | 8.38 | 62.48 | 55.6 | -0.715 |
| YC.08G0021750-3D | CoDof26.3 | 255 | 28689.28 | 5.87 | 54.39 | 56.12 | -0.557 |
| YC.08G0024650-1B | CoDof27.1 | 255 | 28723.29 | 5.87 | 55.02 | 55.73 | -0.545 |
| YC.08G0024650-2C | CoDof27.2 | 255 | 28703.26 | 5.66 | 56.94 | 56.86 | -0.536 |
| YC.08G0024650-3D | CoDof27.3 | 255 | 28675.21 | 5.66 | 54.72 | 55.73 | -0.554 |
| YC.08G0025320-1B | CoDof28.1 | 293 | 31891.46 | 7.56 | 54.14 | 62.83 | -0.557 |
| YC.08G0025320-2C | CoDof28.2 | 308 | 33795.71 | 7.56 | 57.51 | 63.9 | -0.562 |
| YC.08G0025320-3D | CoDof28.3 | 101 | 10919.3 | 4.84 | 26.58 | 70.2 | -0.183 |
| YC.09G0015130-1A | CoDof29.1 | 297 | 32248 | 8.07 | 57.67 | 64.95 | -0.51 |
| YC.09G0015130-2C | CoDof29.2 | 293 | 31959.54 | 8.11 | 55.84 | 62.83 | -0.58 |
| YC.09G0024920-1B | CoDof30.1 | 288 | 31463.38 | 6.39 | 51.48 | 50.17 | -0.765 |
| YC.09G0024920-2D | CoDof30.2 | 226 | 24358.6 | 8.6 | 49.49 | 46.24 | -0.812 |
| YC.10G0009570-1A | CoDof31.1 | 322 | 35058.09 | 6.48 | 44.83 | 48.42 | -0.683 |
| YC.10G0009570-2B | CoDof31.2 | 225 | 24368.68 | 8.6 | 47.74 | 48.62 | -0.804 |
| YC.10G0009570-3C | CoDof31.3 | 126 | 13558.89 | 6.82 | 56.81 | 47.3 | -0.721 |
| YC.10G0009570-4D | CoDof31.4 | 105 | 11423.55 | 9.14 | 65.23 | 42.76 | -0.861 |
| YC.11G0005830-1A | CoDof32.1 | 292 | 32001.9 | 6.55 | 50.91 | 49.49 | -0.795 |
| YC.11G0005830-2B | CoDof32.2 | 322 | 35088.12 | 6.48 | 45.56 | 48.42 | -0.684 |
| YC.11G0005830-3C | CoDof32.3 | 225 | 24342.6 | 8.6 | 48.59 | 46.89 | -0.824 |
| YC.11G0005830-4D | CoDof32.4 | 265 | 28945 | 8.94 | 41.09 | 51.21 | -0.742 |
| YC.11G0027470-1C | CoDof33 | 333 | 35764.65 | 9.21 | 62.3 | 67.09 | -0.603 |
| YC.12G0013310-1A | CoDof34.1 | 333 | 35755.64 | 9.21 | 62.88 | 67.09 | -0.604 |
| YC.12G0013310-2C | CoDof34.2 | 334 | 35892.78 | 9.21 | 62.72 | 66.89 | -0.612 |
| YC.13G0007610-1A | CoDof35.1 | 330 | 35389.26 | 9.21 | 60.44 | 67.7 | -0.576 |
| YC.13G0007610-1P | CoDof35.2 | 347 | 36701.88 | 9.12 | 57.34 | 57.09 | -0.514 |
| YC.13G0007610-2B | CoDof35.3 | 347 | 36744 | 9.12 | 55.52 | 59.34 | -0.483 |
| YC.13G0007610-3D | CoDof35.4 | 347 | 36727.96 | 9.12 | 56.07 | 58.21 | -0.499 |
| YC.14G0008000-1A | CoDof36.1 | 131 | 14766.53 | 9.84 | 56.5 | 38.02 | -1.024 |
| YC.14G0008000-2B | CoDof36.2 | 341 | 36942.24 | 8.72 | 51.7 | 57.24 | -0.56 |
| YC.14G0008000-3C | CoDof36.3 | 340 | 36812.13 | 8.72 | 51.99 | 57.71 | -0.544 |
| YC.14G0008000-4D | CoDof36.4 | 340 | 36828.13 | 8.72 | 52.56 | 57.41 | -0.551 |
| YC.14G0023990-1B | CoDof37 | 133 | 15102.68 | 9.35 | 56.54 | 33.08 | -1.106 |
| YC.15G0002140-1A | CoDof38.1 | 185 | 20583.68 | 9.04 | 57.7 | 38.97 | -0.995 |
| YC.15G0002140-1P | CoDof38.2 | 188 | 20994.98 | 9.04 | 58.91 | 36.28 | -1.105 |
| YC.15G0002140-2C | CoDof38.3 | 185 | 20602.62 | 9.04 | 62.31 | 37.95 | -1.056 |
| YC.15G0002140-2P | CoDof38.4 | 133 | 15038.69 | 9.68 | 57.73 | 33.08 | -1.083 |
| YC.15G0002140-3D | CoDof38.5 | 133 | 15090.65 | 9.54 | 59.85 | 33.08 | -1.111 |
| YC.15G0002140-3P | CoDof38.6 | 320 | 34966.38 | 8.46 | 47.22 | 50.94 | -0.763 |
| YC.15G0007900-1A | CoDof39.1 | 318 | 34809.16 | 7.72 | 48.6 | 51.26 | -0.769 |
| YC.15G0007900-2B | CoDof39.2 | 185 | 20476.46 | 8.85 | 56.9 | 39.51 | -0.997 |
| YC.15G0023810-1C | CoDof40.1 | 318 | 34808.22 | 8.46 | 48.06 | 51.26 | -0.771 |
| YC.15G0023810-2D | CoDof40.2 | 306 | 33008.96 | 8.87 | 58.99 | 57.42 | -0.507 |
