# Supplementary material for: Identification and expression profiling of the CoDof genes involved in fatty acid/lipid biosynthesis of tetraploid Camellia oleifera
Source: Front Plant Sci. 2025 Jun 9;16:1599849. doi: 10.3389/fpls.2025.1599849 (PMC12183095; doi:10.3389/fpls.2025.1599849)
Supplement: Supplementary file 3 [file Table2.docx]

**Table S2.** Cis-regulatory elements annotations.

| **Cis-elements** | **Annotation** | **Annotation Functions** |
| --- | --- | --- |
| GCN4_motif | Cellular Development | cis-regulatory element involved in endosperm expression |
| HD-Zip | Cellular Development | element involved in differentiation of the palisade mesophyll cells |
| MBSI | Cellular Development | MYB binding site involved in flavonoid biosynthetic genes regulation |
| MSA-like | Cellular Development | cis-acting element involved in cell cycle regulation |
| RY-element | Cellular Development | cis-acting regulatory element involved in seed-specific regulation |
| ABRE | Hormone | cis-acting element involved in the abscisic acid responsiveness responsiveness |
| AuxRR-core | Hormone | cis-acting regulatory element involved in auxin responsiveness |
| O2-site | Hormone | cis-acting regulatory element involved in zein metabolism regulation |
| P-box | Hormone | gibberellin-responsive element |
| 3-AF1 binding site | Stress | light responsive element |
| ACE | Stress | cis-acting element involved in light responsiveness |
| AE-box | Stress | part of a module for light response |
| ARE | Stress | cis-acting regulatory element essential for the anaerobic induction induction |
| AT-rich sequence | Stress | element for maximal elicitor-mediated activation (2copies) |
| chs-CMA1a | Stress | part of a light responsive element |
| chs-CMA2a | Stress | part of a light responsive element |
| circadian | Stress | cis-acting regulatory element involved in circadian control |
| ERE | Stress | Ethylene-responsiveelement |
| G-box | Stress | cis-acting regulatory element involved in light responsiveness |
| GC-motif | Stress | enhancer-like element involved in anoxic specific inducibility |
| I-box | Stress | part of a light responsive element |
| LTR | Stress | cis-acting element involved in low-temperature responsiveness |
| MBS | Stress | MYB binding site involved in drought-inducibility |
| MRE | Stress | MYB binding site involved in light responsiveness |
| Sp1 | Stress | light responsive element |
| TC-rich repeats | Stress | cis-acting element involved in defense and stress responsiveness |
| WUN-motif | Stress | wound-responsive element |
| Gap-box | Stress | part of a light responsive element |
| LAMP-element | Stress | part of a light responsive element |
